# Rad4 Mainly Functions in Chk1-Mediated DNA Damage Checkpoint Pathway as a Scaffold Protein in the Fission Yeast *Schizosaccharomyces pombe*

**DOI:** 10.1371/journal.pone.0092936

**Published:** 2014-03-24

**Authors:** Ming Yue, Li Zeng, Amanpreet Singh, Yongjie Xu

**Affiliations:** 1 Department of Biochemistry and Molecular Biology, Boonshoft School of Medicine, Wright State University, Dayton, Ohio, United States of America; 2 Department of Pediatrics, The 1st Affiliated Hospital of Zhengzhou University, Zhengzhou City, China; Florida State University, United States of America

## Abstract

Rad4/Cut5 is a scaffold protein in the Chk1-mediated DNA damage checkpoint in *S. pombe*. However, whether it contains a robust ATR-activation domain (AAD) required for checkpoint signaling like its orthologs TopBP1 in humans and Dpb11 in budding yeast has been incompletely clear. To identify the putative AAD in Rad4, we carried out an extensive genetic screen looking for novel mutants with an enhanced sensitivity to replication stress or DNA damage in which the function of the AAD can be eliminated by the mutations. Two new mutations near the N-terminus were identified that caused significantly higher sensitivities to DNA damage or chronic replication stress than all previously reported mutants, suggesting that most of the checkpoint function of the protein is eliminated. However, these mutations did not affect the activation of Rad3 (ATR in humans) yet eliminated the scaffolding function of the protein required for the activation of Chk1. Several mutations were also identified in or near the recently reported AAD in the C-terminus of Rad4. However, all mutations in the C-terminus only slightly sensitized the cells to DNA damage. Interestingly, a mutant lacking the whole C-terminus was found resistant to DNA damage and replication stress almost like the wild type cells. Consistent with the resistance, all known Rad3 dependent phosphorylations of checkpoint proteins remained intact in the C-terminal deletion mutant, indicating that unlike that in Dpb11, the C-terminus of Rad4 does not contain a robust AAD. These results, together with those from the biochemical studies, show that Rad4 mainly functions as a scaffold protein in the Chk1, not the Cds1(CHK2 in humans), checkpoint pathway. It plays a minor role or is functionally redundant with an unknown factor in Rad3 activation.

## Introduction

Faithful duplication of the genome in eukaryotes depends upon the precise operation of the DNA replication machinery and upon the checkpoint pathways that deal with various perturbations of DNA replication (see reviews [1], [2]). If undetected by the checkpoint, perturbed replication forks become unstable and may undergo catastrophic collapse, resulting in cell death or mutagenic chromosomal damage. Consistent with its important function in maintaining genome integrity, the replication checkpoint is highly conserved from yeast to humans, and defects in the checkpoint pathway are linked to genome instability and cancer [3], [4].

There are two DNA structure checkpoint pathways operating in the fission yeast *S. pombe* [5], [6], [7]. The first pathway is mediated by Chk1 when DNA damage occurs outside of S phase. While arresting the cell in G2, activated Chk1 stimulates DNA repair activities so that the damage can be repaired before the cell undergoes mitosis. The second pathway is the Cds1 (CHK2 in humans) mediated replication checkpoint or intra-S checkpoint that deals with various perturbations of DNA replication. The main function of the activated replication checkpoint is believed to be the protection of perturbed replication forks from collapsing so that the forks can resume with DNA synthesis when the perturbations diminish [8], [9]. Activation of the two checkpoint pathways is normally well coordinated with cell cycle progression. However, the cellular responses stimulated by the two pathways partially overlap such that when one pathway fails, the other is turned on as a backup [10], [11], [12]. For example, when perturbed replication forks collapse in Cds1 mutants, Chk1 is activated to stimulate the repair of the DNA damage resulting from the collapsed forks.

At the apex of the two checkpoint pathways, Rad3-Rad26, Rad9-Rad1-Hus1 (9-1-1 clamp), and Rad17 (9-1-1 clamp loader) function as checkpoint sensors (see reviews [13], [14], [15]). The sensors detect perturbed forks or various types of DNA damage and work in collaboration to activate the effector kinases Cds1 and Chk1 via the mediators Mrc1 [16], [17] and Crb2 [18], respectively. Like ATR in humans and Mec1 in *S. cerevisae*, Rad3 in the Rad3-Rad26 complex is a phosphatidylinositide 3-kinase-related protein kinase (PIKK) [19] and is responsible for the phosphorylation and activation of both Cds1 and Chk1. Rad3-Rad26 associates with replication protein A (RPA) coated single-stranded DNA (ssDNA) to initiate checkpoint signaling [20], [21]. Since ssDNA can be generated by DNA damage and by uncoupling of helicase and polymerases at perturbed forks, Rad3 is activated in response to various DNA lesions and stresses in *S. pombe* [22], [23]. Interestingly, like ATM in humans [24], Rad3 is activated as quickly as can be measured after cells are treated with ionizing radiation [25]. Because of the dynamic fork movement during S phase, the activation of Rad3 at perturbed forks may also follow the unusually fast kinetics. However, despite the remarkable progress in our understanding of checkpoint pathways, the exact mechanism involved in the initiation of checkpoint signaling, particularly at the perturbed forks, remains poorly understood.

Interestingly, several DNA replication proteins also function in the checkpoint signaling. In addition to the aforementioned RPA and Rfc2-6 in the 9-1-1 clamp loader complex Rad17/Rfc2-6 [26], DNA polymerase α and ε have been shown to work in checkpoint signaling in *S. pombe* [27] and *S. cerevisiae* [28], respectively. The replication initiation protein Cdc18 (Cdc6) in *S. pombe* has also been suggested to function in the replication checkpoint [29], probably by anchoring the Rad3-Rad26 complex to chromatin [30]. The exact functions of these multi-functional proteins in the checkpoint pathways remain to be understood.

The *rad4^+^* gene was first reported in *S. pombe* about 40 years ago [31] (see review [32]). It was also identified as *cut5^+^* in a genetic screen of *cut* (cell untimely torn) mutants [33], [34], in which the normal coordination of nuclear division and cytokinesis was disrupted. Four repeated regions in Rad4 were found [18], [35], which are now known as the BRCA1 C-terminus (BRCT) domain. Importantly, Cut5/Rad4 was found to be required for both DNA replication and the DNA damage checkpoint [33]. The homologs of Rad4 were subsequently discovered in budding yeast and in humans as Dpb11 [36] and TopBP1 [37], respectively. Studies in budding yeast have shown that Dpb11 binds to Sld2 and Sld3 that have been prephosphorylated by CDK via its C- and N-terminal pairs of BRCT repeats, respectively, to form a ternary protein complex required for the initiation of DNA replication [38], [39]. A subsequent study showed that this mechanism is conserved in fission yeast Rad4 [40]. Yeast two-hybrid screen has also revealed that Rad4 interacts with Crb2, the mediator for Chk1 activation in *S. pombe* [41]. An earlier study in Xenopus discovered that TopBP1 can directly activate ATR *in vitro* and *in vivo* via its ATR activation domain (AAD) located between the sixth and the seventh BRCT repeats [42]. Subsequent studies in budding yeast showed that the AAD is conserved in the unstructured C-terminus of Dpb11 that can activate Mec1 *in vitro* [43], [44], [45], [46]. A recent study in fission yeast showed that similar to Dpb11, a small AAD of seven amino acids exists in the C-terminus of Rad4 (Lin *et al*. 2012). However, mutations of the AAD in Rad4 only slightly sensitize the cells to DNA damage.

To investigate the potential function of Rad4 AAD in the Cds1-mediated replication checkpoint, we tested several previously reported mutants [31], [33], [47] under the replication stress induced by hydroxyurea (HU), an inhibitor of ribonucleotide reductase. None of the *rad4* mutants showed a high sensitivity. In addition, all previously reported *rad4* mutations appear to affect the scaffolding but not the activation of Rad3. To provide a clear answer, we carried out an extensive genetic screen looking for new *rad4* mutants in which most, if not all, of the checkpoint function is eliminated. Two new mutants C13Y and K56R were identified near the N-terminus that are significantly more sensitive to chronic HU exposure and the DNA damage induced by methyl methanesulfonate (MMS) than all previously reported mutants, suggesting that most of the checkpoint activity is eliminated. However, these mutations did not affect much of the Cds1 activation following HU treatment yet eliminated the scaffolding function of Rad4 required for Chk1, but not Rad3, activation. We also identified several mutations around the recently reported AAD in the C-terminus of Rad4 [48]. However, all C-terminal mutants were minimally sensitive to MMS or HU. Importantly, the mutant with the deletion of the whole C-terminus was resistant to HU and MMS almost like the wild type cells and all known Rad3 dependent phosphorylations were unaffected by the deletion. Together, our results, including those from the *in vitro* biochemical analyses, show that unlike that in Dpb11, the C-terminus of Rad4 does not contain a robust AAD. We believe that in fission yeast, Rad4 plays a minor role or is functionally redundant with an unknown factor in Rad3 activation.

## Materials and Methods

### Yeast culture, PCR and drug sensitivity assay

Growth of *S. pombe* strains (Table S1) and medium preparation followed standard methods [49]. Point mutations were made by Quick-Change mutagenesis PCR using the high fidelity thermal resistant polymerase *pfu* (Agilent Technologies, Santa Clara, CA). All mutations and sequences were confirmed by DNA sequencing (Retrogen, San Diego, CA). Mutational PCRs were performed following the described protocol using the thermal stable *Taq* DNA polymerase [50]. Vectors were introduced into *S. pombe* cells by electroporation. To test the drug sensitivity by spot assay, 2×10^7^ cells/ml of logarithmically growing *S. pombe* were diluted in fivefold steps and spotted onto YE6S plates containing HU or MMS at the indicated concentrations. The plates were usually incubated at 30°C for 3 days and then photographed. Acute drug treatment was performed in liquid cultures containing 2×10^6^ cells/ml. After the drugs were added at the indicated concentrations, small aliquots of the cultures were diluted 1000 fold and then spread on YE6S plates for the cells to recover. After incubation at 30°C for three days, colonies were counted and presented as percentages relative to the untreated cells.

### Phospho-specific and anti-Rad4 polyclonal antibodies

The affinity-purified phospho-specific antibodies against phosphorylated Cds1-Thr^11^, Mrc1-Thr^645^ and Rad9-Thr^412^ were prepared by Bethyl Laboratories, Inc (Montgomery, TX) [51], [52]. The anti-Rad4 polyclonal antibodies were raised in rabbits by Cocalico Biological, Inc (Reamstown, PA) using His-Rad4 purified from *E. coli* as the antigen. The antibodies, purified from the anti-serum using GST-Rad4(Δ498–648aa) as the antigen, were used for the Western blotting analyses.

### Immunoprecipitation (IP), Co-IP, and Western blotting

The phosphorylation of hemagglutinin (HA) epitope tagged Cds1, Rad9 and Crb2 was assessed by Western blottings using phospho-specific antibodies in proteins IPed with anti-HA antibody beads (Santa Cruz Biotechnology, Inc.). Cell lysates were made by a mini-bead beater with acid-washed glass beads in 2× IP buffer (50 mM HEPES/NaOH, pH 7.5, 2 mM Na_3_VO_4_, 20 mM NaP_2_O_7_, 100 mM NaF, 120 mM glycerol phosphate) containing protease inhibitors. After clarification by centrifugation at 4°C, 13,000 rpm (F45-24-11 rotor, Eppendorf 5414R centrifuge) for 5 min, the supernatants were incubated with anti-HA antibody beads by rotating at 4°C for 2 h. After extensive washes with Tris-buffered saline containing 0.5% Tween-20 (TBS-T), the protein was used for *in vitro* binding reactions or separated by SDS-PAGE for Western blotting analyses. The loading of Cds1, Rad9 and Crb2 was monitored first by Western blotting using anti-HA antibody. The same blots were stripped and reprobed with phospho-specific antibodies for detection of the site-specific phosphorylation of the proteins. Phosphorylated Chk1 was assessed by mobility shift assay using anti-HA antibody [25]. The immunoblotting signal was detected by chemiluminescence and photographed using the Image Reader LAS-3000 (Fuji Film, Inc.). Intensities of the bands were analyzed by ImageGauge software (Fuji Film, Inc.).

### Protein purifications

GST tag was fused with Rad4 fragments at the N-terminal ends for expression and purification in *E. coli*. The recombinant proteins were induced to express in BL21(DE3) cells by incubation at 30°C for 4 h after isopropyl 1-thio-D-galactopyranoside was added to 0.4 mM. The *E. coli* cells were harvested and lysed in a cell homogenizer (Avestin, Inc.) in 20 mM phosphate buffer (pH 7.5) containing 500 mM NaCl, 1 mM DTT, 0.2% Tween-20, 0.2% NP-40, and protease inhibitors. Cell lysate was clarified by centrifugation at 4°C, 13,000 rpm (F45-24-11 rotor, Eppendorf 5414R centrifuge) for 30 min. The supernatant was loaded onto a GSTrap column (GE Healthcare). After extensive washes with the lysis buffer, the proteins were eluted in the lysis buffer containing 10 mM glutathione. The eluted proteins were dialyzed overnight against the binding buffer (25 mM Tris-HCl, 150 mM NaCl, 1 mM DTT, 0.02% Tween-20, 0.02% NP-40, 10% glycerol, and 1 μM leupeptin, pH 7.5). Full-length Rad4 was fused with GST at the N-terminus and overexpressed in *S. pombe* under the control of *nmt1^+^* promoter. The cell lysate was made by grinding the frozen cells in a coffee-mill with dry ice. The clarified supernatant was loaded onto the GSTrap column, washed, and eluted as described above. While dialyzing overnight, the GST tag was cleaved off with C3 protease. The cleaved full-length Rad4 was directly used without further purification.

The Rad3-Rad26-Flag complex was overexpressed in Δ*rad3*Δ*rad26 S. pombe* under the *nmt1^+^* (Rad3) and *nmt41^+^* (Rad26-Flag) promoters. The yeast cells were homogenized in 2× IP buffer by the cell homogenizer. The clarified cell extract was incubated with anti-Flag M2 antibody beads (Sigma) by rotating at 4°C for 2 h. The beads were washed five times in ice-cold TBS-T buffer and the proteins eluted in TBS-T buffer containing 0.1 mg/ml Flag peptide for 60 min at 4°C. The kinase-inactive Rad3(D2249E)-Rad26-Flag complex [53] was purified under the similar conditions.

### *In vitro* binding assay

Each purified Rad4 fragment was incubated with HA-Rad9 or HA-Crb2 bound to anti-HA antibody beads at a final concentration of 0.5 μM by rotating at 4°C for 2 h in 100 μl binding buffer (20 mM TrisHCl, 150 mM NaCl, 0.02% NP-40, 1 mM DTT and 10% glycerol, pH 7.5). After washing several times with the binding buffer, the bound proteins were eluted with SDS gel loading buffer followed by heating at 98°C for 10 min. The samples were analyzed by SDS-PAGE or Western blotting.

### *In vitro* Rad3 kinase assay

To assess the kinase activity of Rad3-Rad26, the kinase-inactive Cds1(D312E) mutant protein purified from *E. coli*. was used as the substrate [52]. The kinase reactions were carried out in a total volume of 15 to 30 μl with the standard kinase reaction buffer (50 mM Tris-HCl, 10 mM MgCl_2_, 2 mM DTT, 0.1 mM EDTA, 0.01% NP-40, 200 μM ATP, pH 7.5) containing 500 ng of the Cds1(D312E) substrate. The reactions were assembled on ice and started by incubating at 30°C. To stop the reactions, the samples were chilled on ice and immediately mixed with the SDS gel-loading buffer. All samples were examined by Western blotting analyses. Loading of the substrate Cds1(D312E) was shown by Ponceau S staining. Phosphorylation of Cds1-Thr^11^ was examined by the phospho-specific antibody on the same membrane. Anti-HA and anti-Flag antibodies were used to detect Rad3 and Rad26, respectively.

## Results

### Identification of novel *rad4* mutants with enhanced sensitivity to HU and MMS

**Figure 1:**
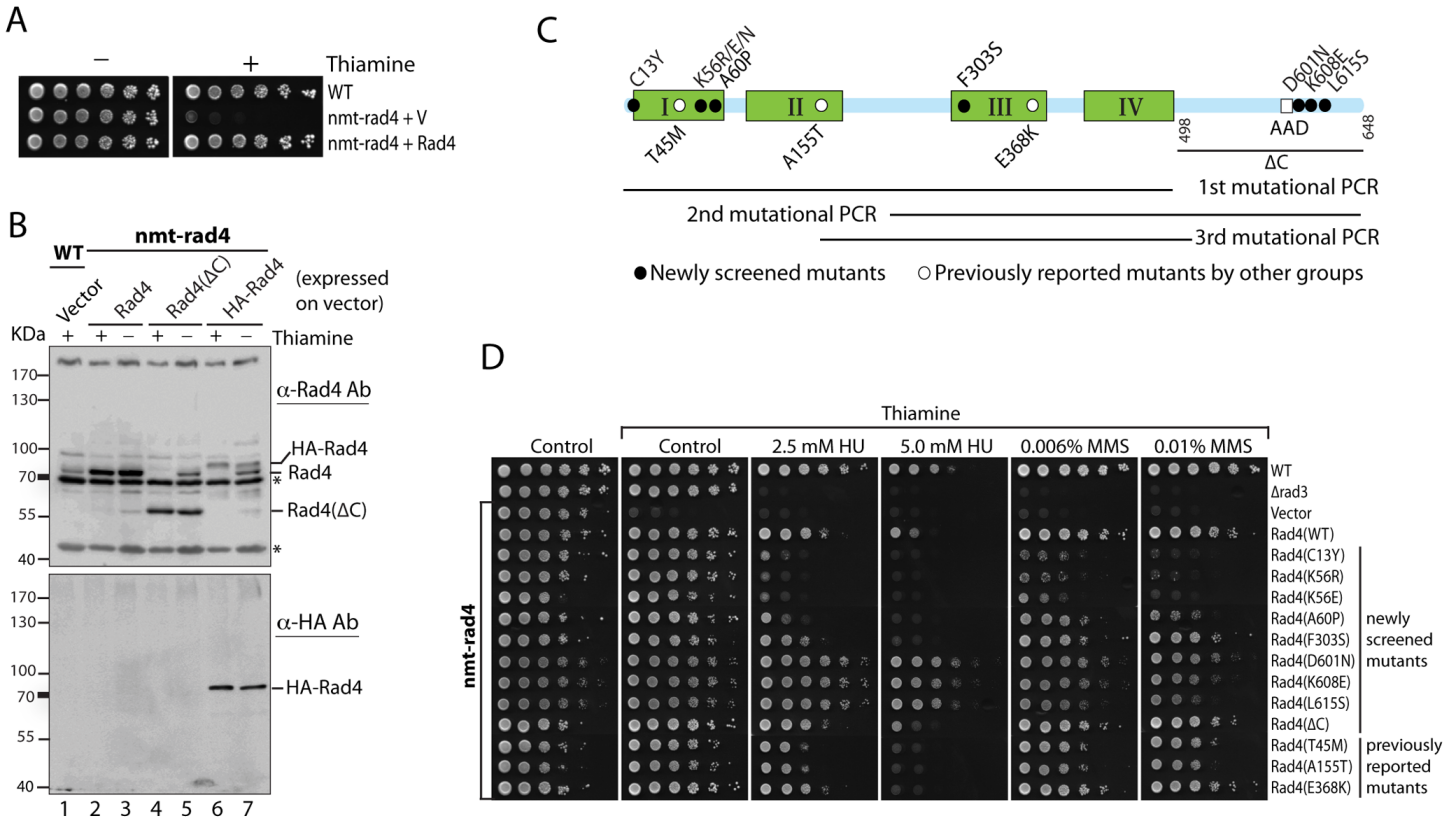
Genetic screen of novel *rad4* mutants with enhanced sensitivities to HU and MMS in the *rad4^+^* shut-off strain. **A**, thiamine-controlled cell growth of the shut-off strain (nmt-rad4) in which the endogenous promoter of *rad4^+^* was replaced with a thiamine repressive *nmt81* promoter. Logarithmically growing cells were diluted in fivefold steps and spotted on EMM6S plates with (+) or without (-) thiamine. The plates were incubated at 30°C for 3 days before being photographed. **B**, thiamine-regulated expression of Rad4 was examined by Western blotting. Untagged Rad4, Rad4 with the deletion of whole C-terminus (ΔC) and HA-tagged Rad4 were expressed on a vector in the shut-off strain. Expression of Rad4 in the presence (+) or absence (-) of thiamine was examined by using anti-Rad4 antibodies (Top). The same membrane was stripped and blotted with anti-HA antibody (bottom). Asterisks indicate the cross-reacting materials. **C**, molecular architecture of Rad4 with relative locations of the newly isolated (solid circles) and previously reported (open circles) mutations and the AAD domain. The four BRCT repeats are marked by roman numerals. The regions covered by mutational PCRs in the genetic screening are also shown. **D**, sensitivities of the *rad4* mutants to HU and MMS were assessed by spot assay in the shut-off strain. The newly screened (top part) and the previously reported (lower part) mutations are marked on the right. Wild type cells, Δ*rad3* mutant, and the shut-off strain carrying an empty vector or the vector expressing wild-type Rad4 were used as controls.

**Figure 2:**
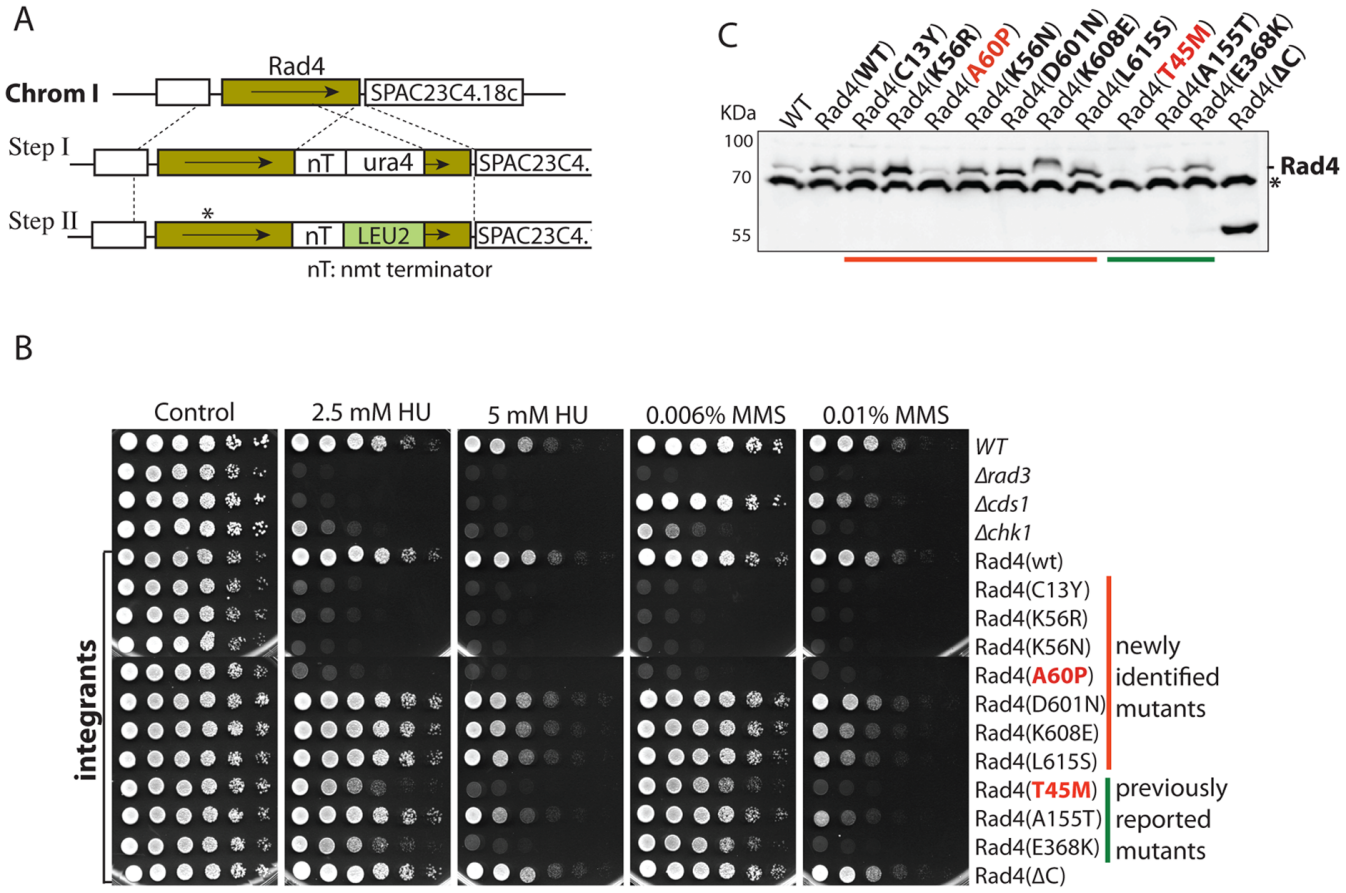
Drug sensitivities of *S. pombe* with the integrated *rad4* mutations. **A**, the two-step marker switching method used for integration of the mutations. The genomic locus of *rad4^+^* is shown on top. **B**, sensitivities of the cells with integrated *rad4* mutations to HU or MMS were examined by spot assay. The newly identified (upper part) and the previously reported (lower part) mutants are marked on the right. **C**, expression of Rad4 in the cells with the integrated *rad4* mutations was detected in whole cell lysates by Western blotting using anti-Rad4 antibodies. The asterisk denotes the cross-reactive material. Note: like the T45M mutant, A60P is a *ts* mutant.

In addition to its roles in DNA replication and the DNA damage checkpoint as a scaffold protein, previous studies have suggested that Rad4 may also function in the replication checkpoint mediated by Cds1 [33], [47], [54]. However, none of the previously reported *rad4* mutants showed a high sensitivity to the replication stress induced by HU (see lower parts in Fig. 1D and 2B). Some of the previous studies also used temperature sensitive (*ts*) mutants, which may cause an unnoticeable defect in DNA replication or other functions. In order to provide a clear answer, we decided to screen new *rad4* mutants with an enhanced sensitivity to replication stress. To facilitate the screening, we made a shut-off strain in which the promoter of *rad4^+^* at the genomic locus was replaced with a thiamine repressive *nmt81* promoter so that expression of Rad4 can be switched off by adding thiamine to the medium. The essential replication function is supported by wild type or mutant Rad4 expressed on a vector. As shown in Fig. 1A, the shut-off strain (nmt-rad4) carrying an empty vector behaved normally in the absence of thiamine but could not grow in the presence of thiamine. The same strain with a vector expressing Rad4 grew normally with or without thiamine. To monitor protein expression, Western blotting was performed with whole cell lysates using anti-Rad4 antibodies (Fig. 1B). Untagged Rad4, Rad4 with an N-terminal HA tag, and the Rad4 mutant lacking the whole C-terminus (ΔC) were expressed on the vector. The endogenous Rad4 was detected in wild type cells as well as in the shut-off strain growing in the absence of thiamine (compare lane 1 with lanes 3, 5, and 7). In the presence of thiamine, the endogenous Rad4 was undetectable in the shut-off strains expressing Rad4(ΔC) or HA tagged Rad4 on the vector, which have different migration rates than untagged Rad4 (compare lanes 1 and 2 with lanes 4 and 6). These data confirmed the tight regulation of the endogenous Rad4 expression and showed that the essential replication function can be fully supported by extrachromosomally expressed Rad4 or the Rad4(ΔC) mutant.

Once the shut-off strain was made available, random mutations were generated in *rad4* by first two rounds of mutational PCRs [50] that cover the full-length of the protein (Fig. 1C). The mutated proteins were expressed in the shut-off strain on a vector with LEU2 marker. The *leu^+^* colonies grown on plates without thiamine were replicated onto plates containing thiamine alone or thiamine plus HU or MMS. The thiamine alone plates were used to remove mutants with defects in DNA replication. The checkpoint mutants were isolated by comparing the colonies on plates containing thiamine alone with those on the plates containing thiamine plus the drugs. After extensive screenings and removal of the replication and the by-stander mutations, seven new checkpoint mutations were identified that are distributed across the molecule (Fig. 1C, black dots). Among the mutated residues, K56 was most frequently mutated (∼4 fold higher), suggesting the importance of the residue in checkpoint. To confirm the newly identified mutants, all mutations were remade by point mutational PCRs, confirmed by DNA sequencing, and tested in the shut-off strains for sensitivities to HU or MMS (Fig. 1D). For comparison, three previously reported mutations T45M, A115T and E368K [31], [33], [47] were also made and tested by the same method (Fig. 1D, lower part). We found that each of the four newly identified mutations C13Y, K56R, K56E and A60P in the first BRCT repeat caused more dramatic HU and MMS sensitivities than that in the previously reported mutants. Interestingly, all mutations found at the K56 residue K56R, K56E, and K56N had a similar drug sensitivity (see Fig. 1 and below), indicating that the conformation, not the charge, of this residue is critical for the checkpoint function. The newly identified F303S mutation is located in the third repeat as is the previously reported E368K mutation [47]. Consistently, it sensitized the cells to a similar degree as the E368K mutation. Interestingly, the overall patterns of the relative sensitivities of all *rad4* mutants to HU and MMS are quite similar, suggesting that Rad4 mainly functions in the same Chk1-mediated DNA damage checkpoint pathway (see below).

### Similar drug sensitivities caused by the integrated *rad4* mutations

To avoid the issues of plasmid loss or protein overexpression, we integrated the newly screened mutations at the *rad4^+^* genomic locus by a two-step marker switching method (diagramed in Fig. 2A). In the first step, an expression cassette consisting of the open reading frame of *rad4^+^*, the *nmt1^+^* terminator and *ura4^+^* marker was used to replace *rad4^+^* at the genomic locus. Colony PCR and back-crosses with a wild-type strain proved the correct replacement. In the second step, the mutant *rad4* carrying the *LEU2* marker was integrated in a diploid strain with one copy of *rad4^+^* has been linked to the *ura4^+^* marker in the first step. The *ura^−^/leu^+^* diploid colonies were screened by colony PCR and subsequent sequencing confirming the integrated mutations. Diploid strains were allowed to sporulate to generate *leu^+^* haploid cells carrying the integrated mutations. For comparison, the three previously reported mutations T45M, A155T and E368K were also integrated by the same method. The F303S mutation was not integrated because it resides in the same BRCT repeat and had similar drug sensitivities as the E368K mutant [47]. Consistent with the results shown in Fig. 1D, none of the integrated mutations affected the cell growth much suggesting that the DNA replication function of the protein is not or minimally affected.

The drug sensitivities of *S. pombe* with the integrated mutations were reassessed by spot assay (Fig. 2B). Similar to Fig. 1D, the C13Y, K56R, K56N and A60P mutants were significantly more sensitive to MMS and HU than the three previously reported mutants. These new mutants, especially the C13Y, K56R and K56N mutants, showed a similar or even higher sensitivity than cells lacking Chk1, indicating that most, if not all, of the checkpoint function is eliminated by the mutations. However, all these mutants were less sensitive than the Δ*rad3* mutant (see below), indicating that Rad4 functions below Rad3 in the checkpoint pathways. Interestingly, the ΔC mutant with the deletion of the whole C-terminus between amino acid 498 and 648 was resistant to HU and MMS almost like the wild type cells (Fig. 1D and 2B). The only difference we could readily find for the ΔC mutant was that the protein level was higher than in the wild type cells (Fig. 2C), suggesting that the C-terminus may not contain a robust AAD (see below).

Considering that some of the mutations may affect protein stability, we examined the protein levels in cells with the integrated *rad4* mutations (Fig. 2C). Most of the mutant proteins were stable except the A60P, which was at a lower level similar to the known T45M *ts* mutant [31], [33]. To see whether some of the mutants are *ts*, we tested the growth of those mutants with significant drug sensitivities under different temperatures. All mutants grew well at both 37°C and 25°C, except the A60P mutant, which was only able to grow at 25°C (Fig. S1). Since *ts* mutations may complicate the experiments by minimally affecting DNA replication, we did not follow up the A60P mutant in the rest of this study.

### The C13Y and K56R mutations may have eliminated most of the checkpoint function of Rad4

Since deletion of Rad4 C-terminus did not sensitize the cell to DNA damage and the replication stress, we surmised that a robust AAD may exist in the N-terminal BRCT repeat region. The higher drug sensitivities of all mutants in this region suggest this possibility. However, an ideal mutant that can completely eliminate the checkpoint function of Rad4 may remain to be identified. If activation of Rad3 by Rad4 is absolutely required for the checkpoint signaling, discovery of such an ideal mutant should provide a definite answer. To search for this ideal mutant, we tried to combine some of the point mutations that cause severe drug sensitivities (Fig. S2). We found that combinations of the previously reported E368K mutation [47] with K56R or F303S were lethal suggesting a defect in DNA replication. Combination of C13Y and K56R mutations with the mutations in C-terminus did not further increase the drug sensitivity (data not shown). Although C13Y can be combined with K56R or E368K, no significant increase in drug sensitivity was observed. Together, these results suggest that the N-terminal C13Y, K56R or C13Y-K56R mutations may have maximally separated the functions of Rad4 in checkpoint and DNA replication.

### The C13Y and K56R mutations eliminated the activation of Chk1, but not Cds1, by Rad3

**Figure 3:**
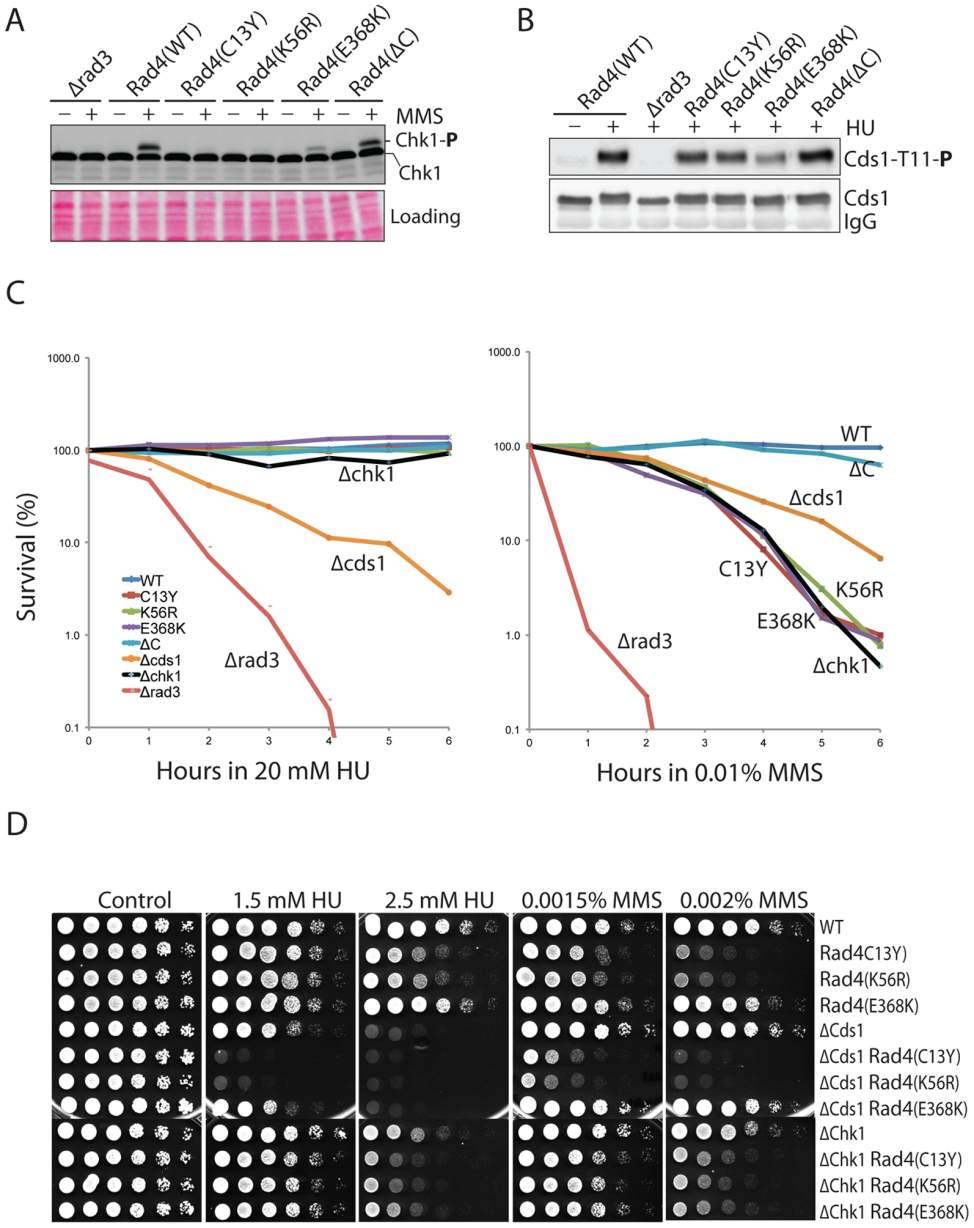
Rad4 mainly functions in Chk1-mediated DNA damage checkpoint pathway. **A**, wild type cells or cells with the integrated mutations were treated with (+) or without (−) MMS at 30°C for 1 hour. Phosphorylation of Chk1 was monitored by the mobility shift assay as described in the Materials and Methods. A section of the Ponceau S stained membrane is shown for the loading (bottom). **B**, Rad3-dependent phosphorylation of Cds1-Thr^11^ was examined by phosphor-specific antibody (top panel). Loading of Cds1 is shown in the lower panel. **C**, sensitivity of the cells to acute HU (left) or MMS (right) treatment. Cells were treated with the drugs in YE6S medium at 30°C. At each time point, an aliquot of the culture was removed and spread on YE6S plates for the cells to recover. Colonies were counted and viability was presented as percentages of the untreated cells. Each data point represents an average of three independent experiments for each mutant. **D**, synthetic effects of the double mutants containing Δ*cds1* or Δ*chk1* with the indicated *rad4* mutations were examined by spot assay.

Since the new N-terminal mutants are highly sensitive to HU and MMS (Fig. 2B), we examined the activation of Chk1 and Cds1 by monitoring their Rad3-dependent phosphorylation using the established methods [25], [51], [55]. Because the K56E and K56N mutants behaved the same as the K56R mutant, we focused on the K56R mutant only. The previously reported E368K mutant [47] was used in the following studies as a control and as a representative of the mutations in the second pair of BRCT repeats. We found that the C13Y and K56R mutations completely eliminated the phosphorylation of Chk1 in MMS-treated cells (Fig. 3A), consistent with their dramatic sensitivity to DNA damage. However, when treated with HU, phosphorylation of Cds1-Thr^11^ by Rad3 in the two mutants was only slightly decreased (Fig. 3B), indicating that the activation of Cds1 was minimally affected by the mutations. The slight decrease in Cds1 phosphorylation may be caused indirectly by a minor defect in DNA replication [56], [57]. This result was a surprise because these mutants are highly sensitive to HU (Fig. 2B) and activation of Cds1 is absolutely required for the checkpoint response to replication stress. We then examined the phosphorylation of Mrc1-Thr^645^, the more proximal substrate, by Rad3 and found that Mrc1 phosphorylation also remained largely intact (Fig. S3). These results showed that in the presence of acute HU treatment, the signaling from Rad3 to Mrc1 and Cds1 was essentially unaffected by the C13Y and K56R mutations. In contrast and consistent with the previous report [47], the E368K mutation partially affected the phosphorylation of both Cds1 and Chk1 (Fig. 3A and 3B). To rule out the indirect cell cycle effect, phosphorylation of Cds1 and Chk1 was examined every hour in a 5 h long experiment (Fig. S4). Similar results were observed. However, it remains possible that the lower levels of Chk1 and Cds1 phosphorylations are caused indirectly by a minor defect in DNA replication [56], [57]. Consistent with the drug resistance, the ΔC mutation did not affect the Rad3 dependent phosphorylation of Chk1, Cds1-Thr^11^, Mrc1-Thr^645^ (Fig. 3A and B, S3) and Rad9-Thr^412^ (see below).

To understand why Cds1 activation is only minimally affected in the C13Y and K56R mutants, we examined their sensitivities to acute HU treatments (Fig. 3C). We found that unlike the Δ*rad3* or Δ*cds1* cells, the C13Y, K56R and E368K mutants remained resistant or slightly sensitive to HU like the Δ*chk1* mutant even after 6 h of treatment. However, under similar conditions, all three *rad4* mutants were sensitive to MMS like the Δ*chk1* mutant. These results suggest that Rad4 mainly works in the Chk1-mediated DNA damage checkpoint pathway. Since all tested *rad4* mutants were less sensitive than Δ*rad3* mutant, Rad4 functions below Rad3.

In fission yeast, when replication checkpoint fails to function properly, Chk1 is activated to stimulate the repair of DNA damage generated from the collapsed forks. However, monitoring the checkpoint activities during a chronic HU treatment is technically challenging because of the weak signals and uncertainties in the amount of drug decomposing over time and the specific time points which would best reflect the real situation inside the dying cells. To investigate why the *rad4* mutants are sensitive to HU only in chronic exposure, we crossed the mutations into Δ*cds1* and Δ*chk1* strains and examined the possible synthetic effects (Fig. 3D). The double mutants containing Δ*cds1* and C13Y or K56R were much more sensitive to HU or MMS than the single mutants indicating a synthetic effect. In contrast, combination of Δ*chk1* with each of the *rad4* mutations did not further increase the drug sensitivity much. These results showed that Rad4 functions primarily in the same pathway with Chk1, not Cds1. The chronic HU sensitivity of the N-terminal *rad4* mutants observed in the spot assay was likely due to the defect in Chk1 activation exacerbated by a possible minimal defect in DNA replication.

### The C13Y and K56R mutations abolished the scaffolding function of Rad4 required for the activation of Chk1 but not Rad3

**Figure 4:**
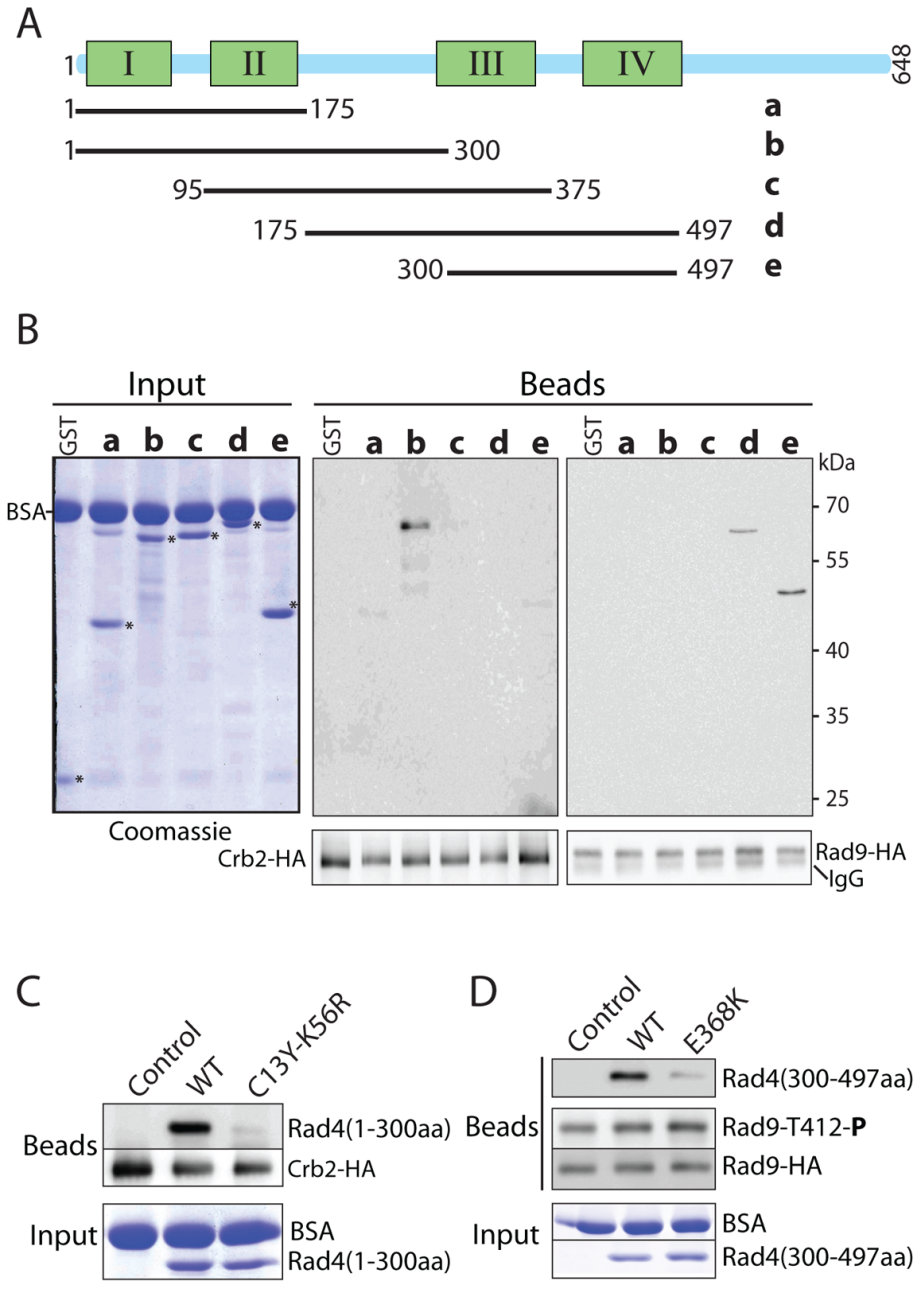
The C13Y and K56R mutations abolish the scaffolding function of Rad4 *in vitro*. **A**, schematic diagram of the Rad4 fragments used in this experiment. Each fragment was fused with a GST tag for protein purification and detection in the binding assay using anti-GST antibodies. **B**, preferential binding of the N- and C-terminal pair of BRCT repeats of Rad4 to Crb2 and Rad9, respectively. Recombinant GST or GST-tagged Rad4 fragments were incubated with immunopurified Crb2 (middle panels) or Rad9 (right panels) bound to the anti-HA antibody beads. The bound proteins were released from the beads in gel loading buffer and analyzed by Western blotting. The membrane was striped and reprobed with anti-HA antibody for Crb2 and Rad9 (bottom panels). Aliquots of the binding reaction were analyzed separately by SDS PAGE as the input (left panel). **C**, equal amount of the Rad4 fragment b with or without the C13Y-K56R mutation was incubated with Crb2 bound on beads. The input and the Rad4 protein bound to Crb2 were analyzed as in B. **D**, fragment e with or without the E368K mutation was similarly tested for binding to phosphorylated Rad9. Phosphorylation of Rad9-Thr^412^ was detected by blotting of the same membrane with the phospho-specific antibody.

Since the Rad3 dependent phosphorylation of Mrc1-Thr^645^, Cds1-Thr^11^ and Rad9-Thr^412^ (see below) was unaffected by the C13Y and K56R mutations, it is unlikely that these mutations affected the activation of Rad3. Previous studies mainly by yeast two hybrid and Co-IP methods showed that Rad4 interacts with Crb2 and Rad9 via its N- and C-terminal pairs of BRCT repeats, respectively [18], [47], [58]. Since the new highly sensitive mutants were available, we repeated the experiments by examining the direct interactions of Rad4 with Rad9 and Crb2. Five Rad4 fragments covering the four BRCT repeats were fused with GST and purified to apparent homogeneity (a to e in Fig. 4A). The purified fragments were incubated at equal amounts of Crb2 or Rad9 immunopurified from MMS or HU treated cells. After extensive washes, Rad4 fragments bound to the anti-HA antibody beads were examined by Western blotting with anti-GST antibodies. As shown in Fig. 4B, fragment b containing the first pair of BRCT repeats, bound preferentially to Crb2, not Rad9. In contrast, fragments d and e containing the second pair of repeats preferentially interacted with Rad9 although a weak binding of fragment e to Crb2 could also be detected. Fragment c, which contains the second and third repeats did not bind to any of the proteins, suggesting that they do not form a functional pair. These results confirmed the data from previous studies [18], [47], [58] and provided a method for assessing the effect of the newly identified mutations on protein-protein interactions. For this purpose, fragment b containing the C13Y-K56R mutation was purified and included in the binding reaction. The C13Y and K56R mutations have been shown to function as a single unit (Fig. S2A). As compared with the wild-type fragment, the binding of the mutant fragment to Crb2 was dramatically reduced (Fig. 4C). Similarly, the E368K mutation eliminated the binding of fragment e to phosphorylated Rad9 (Fig. 4D).

**Figure 5:**
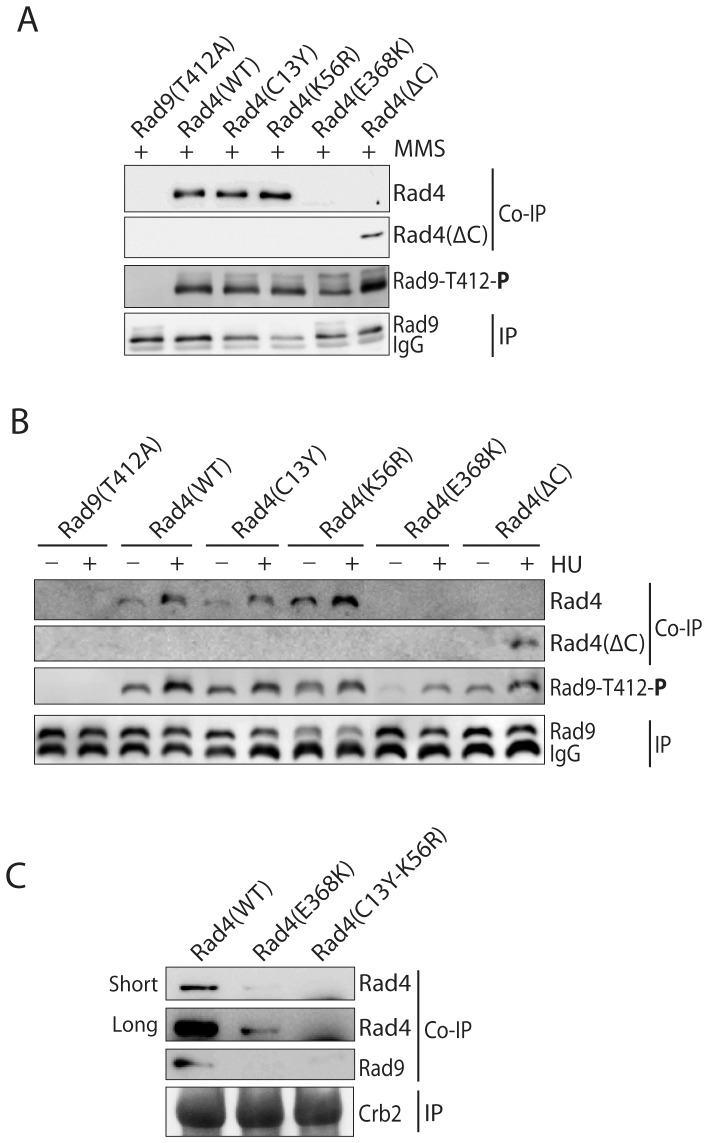
The C13Y and K56R mutations abolish the scaffolding function of Rad4 *in vivo*. **A**, Rad9 was IPed from the lysates of MMS-treated cells with the indicated mutation (bottom panel). Wild type or mutant Rad4 (top two panels) Co-IPed with Rad9 was detected by Western blotting using anti-Rad4 antibodies. Phosphorylated Rad9-Thr^412^ was shown in the third panel from the top. **B**, Co-IP of Rad4 containing the indicated mutations with phosphorylated Rad9 from HU-treated cells. **C**, Co-IP of Rad4 and Rad9 with Crb2 from MMS-treated cells.

To see whether these mutations affect the protein-protein interactions *in vivo*, we performed the Co-IP experiments. Consistent with the *in vitro* results, the E368K mutation abolished the binding to Rad9 as previously reported [47] (Fig. 5A and B) whereas the C13Y-K56R mutation abolished the interaction with Crb2 (Fig. 5C), not Rad9 (Fig. 5A and B). Consistent with the previous report [58], the interaction between Rad9 and Rad4 was dependent on Rad9 phosphorylation because the phosphorylation site mutant Rad9-T412A could not pull-down Rad4 (Fig. 5A, first lane on the left) and the interaction between Rad9 and Rad4 was sensitive to λ-phosphatase treatment (Fig. S5A). As mentioned above, the ΔC mutation did not affect the interaction with Rad9 (Fig. 5A and B) and phosphorylation of Rad9-Thr^412^ by Rad3 (Fig. 5A and B). Together, these *in vitro* and *in vivo* data show that Rad4 mainly function in scaffolding Crb2 and Rad9 for efficient activation of Chk1 by Rad3, not the activation of Rad3.

Since the E368K mutation affected the phosphorylation of both Cds1 and Chk1 (Fig. 3A and B), we suspected that the second pair of BRCT repeats may contain a robust AAD for Rad3 activation. However, the recruitment of Rad4 requires prephosphorylation of Rad9 by Rad3, which is inconsistent with the possibility. To investigate further, we crossed the E368K mutant into the Rad9 phosphorylation site mutants T412A and T412A-S423A [58] and examined the genetic relationship between the recruitment of Rad4 and the phosphorylation of Rad9 (or Rad3 activation). We found that the Rad9 phosphorylation site mutations had a stronger effect than the E368K mutation on the phosphorylation of Cds1-Thr^11^ by Rad3 (Fig. S5B) and the resistance to MMS (Fig. S5C). This result suggests that Rad4 recruited by phosphorylated Rad9 promotes the phosphorylation of Chk1 by Rad3 but not the activation of Rad3. It is also consistent with our previous data that deletion of *rad9* or mutations of Rad9 phosphorylation sites do not affect the phosphorylation of Mrc1 by Rad3 [51]. The third mutational PCR screening focusing on the second pair of BRCT repeats in Rad4 also supports the idea that this region does not contain a robust AAD (see below). We believe that the recruited Rad4 is required for amplification of the checkpoint signaling pre-initiated by Rad3 [48]. Since immunopurification of endogenous Rad3 for direct measurement of the kinase activity remains technically challenging, the exact mechanisms of how Rad9 is phosphorylated by Rad3 for recruiting Rad4 and subsequent amplification of the signaling remains unknown.

### The C-terminus of Rad4 does not contain a robust AAD

Like the AAD originally identified in TopBP1, the C-terminus of Dpb11 can robustly stimulate Mec1-Ddc2 kinase activity in *S. cerevisiae* [43], [44], [45]. Three new mutations D601N, K608E and L615S were isolated by our screening in or near the small AAD recently identified in the C-terminus of *S. pombe* Rad4 (Fig. S6A) [48]. However, these mutations, including the Y599R [48] and D601N in the AAD, only caused a minimal sensitivity to MMS and HU (Fig. S6B). Since the K608E and L615S mutants showed a similar sensitivity as theY599R mutant, the aromatic resides may not be required for the function of the putative AAD [42], [44]. Consistent with this, mutations of the only two tryptophans W485 and W580 in the C-terminus and a third tryptophan W253 based on the similarity of the local sequences between Dpb11 and Rad4 did not sensitize the cells (Fig. S7D).

Since all AAD mutants in Rad4 C-terminus were mildly sensitive to DNA damage, it is possible that the genetic screen was not exhaustive and failed to identify the true AAD. To search for this domain in the C-terminus, we made a series of deletions from the C-terminal end (Fig. S7A, C and E). Among the deletions, only the Δ610–648 near the AAD caused a minimal MMS sensitivity similar to that of the AAD mutants. The (Δ474–648) mutant with a larger deletion than ΔC grew normally with no observed MMS sensitivity. However, the mutant was slightly sensitive to HU, suggesting a minor defect in replication. Further deletion into the fourth BRCT repeat is lethal. Together, these results provide further support to the notion that Rad4 C-terminus does not contain a robust non-redundant function for Rad3 activation. The minimal drug sensitivity of the C-terminal mutants suggests that either the C-terminus can activate Rad3 under certain specific situations [48] or the drug sensitivity is caused indirectly by a secondary effect. Consistent with the latter possibility, the extrachromosomally expressed C-terminal mutants, including the Y599R mutant, in the shut-off strain were more resistant to HU than wild type cells (Fig S6C), suggesting that they may promote DNA replication under stress. Furthermore, under the moderate replication stress induced by 2.5 mM HU, this effect of the C-terminal mutations can even suppress the checkpoint defect of the N-terminal C13Y and K56R mutations (Fig. S6C). When cells were treated with MMS or a high dose of HU to generate DNA damage, the N-terminal mutations were dominant over the C-terminal mutations (Fig. S6C).

### Minimal effect of purified Rad4 on the kinase activity of inactive Rad3-Rad26 *in vitro*

Studies in other model organisms strongly suggest that Rad4 may contain a robust AAD that can directly activate Rad3 *in vivo* and *in vitro* [42], [43], [44], [46]. A recent study found a small AAD of seven amino acids in the C-terminus [48]. Mutations in the AAD slightly sensitize the cells to DNA damage and eliminate the activity of Rad3 in an assay with induced recruitment of checkpoint proteins to chromatin. Our genetic screen also identified mutations in or near the AAD with minimal drug sensitivities. However, the results described above showed that Rad4 C-terminus may not contain a robust AAD and that the main function of Rad4 is to scaffold checkpoint proteins for efficient activation of Chk1, not the activation of Cds1 or Rad3. It is possible that our genetic screen is not exhaustive and the true AAD that can robustly activate Rad3 remains to be identified in other parts of Rad4. To investigate this possibility, we carried out a third round screen focusing on the linker between the two BRCT pairs and the third BRCT repeat because the E368K mutation in this region has a general impact on both Cds1 and Chk1 pathways (Fig. 1C). However, none of the screened mutants were more sensitive than the F303S and E368K mutants, suggesting that a robust AAD may not exist in this region and the function of this region may be more tightly linked to DNA replication. Another possibility is that the robust AAD is genetically inseparable from the essential replication function.

**Figure 6:**
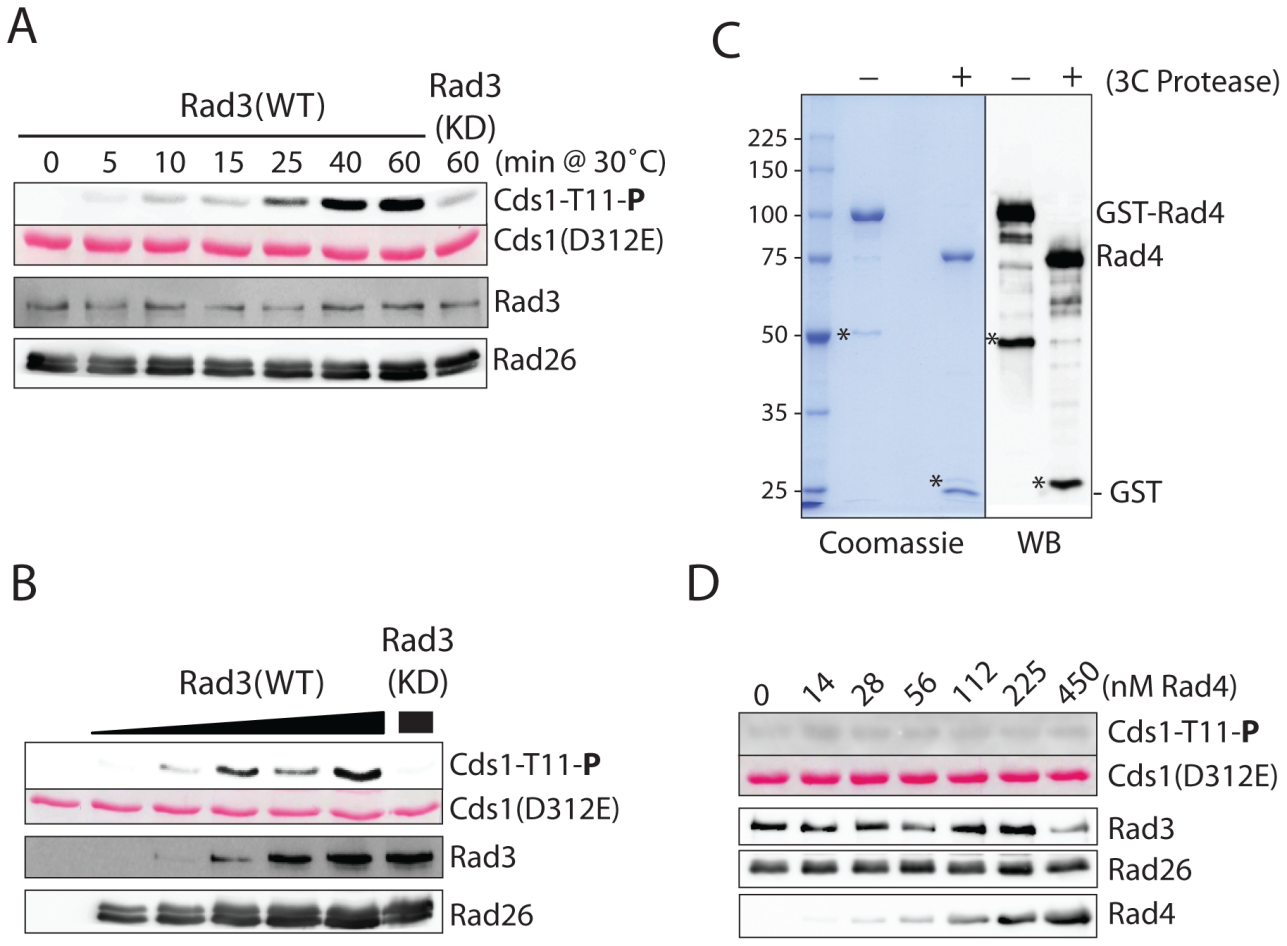
*In vitro* kinase assay using purified Rad3-Rad26 and Cds1(D312E) as the substrate. **A**, time course of the *in vitro* kinase reaction. Rad3-Rad26 was affinity-purified from *S. pombe* using anti-Flag antibody resin, eluted with Flag peptide, and incubated with the kinase-inactive Cds1(D312E) as the substrate in the standard kinase buffer containing 200 μM ATP. At each time point, a small aliquot of the reaction was taken out and analyzed by SDS PAGE followed by Western blotting to examine the phosphorylation of Cds1-Thr^11^. Rad3 and Rad26 in the same samples were detected by using anti-HA and anti-flag antibody, respectively. Loading of the substrate was shown by Ponceau S staining. The kinase dead Rad3(D2249E)-Rad26 was similarly prepared and used as the control (last lane on the right). **B**, phosphorylation of Cds1-Thr^11^ was examined in the presence of increasing concentrations of Rad3-Rad26. The reaction was carried out at 30°C for 30 min. **C**, full-length Rad4 was purified from *S. pombe*. Removal of the N-terminal GST tag was analyzed by SDS PAGE (left) and confirmed by Western blotting (right) using anti-Rad4 antibodies. Asterisks indicate the degradation products of Rad4. **D**, the Rad3 kinase reactions were carried out at 30°C for 20 min in the absence or presence of increasing amount of purified Rad4 and analyzed as in A.

To investigate this possibility, we purified full-length Rad4 from *S. pombe* (Fig. 6C) and assessed its direct effect on the kinase activity of Rad3 under similar conditions described in the previous studies with Mec1 [43], [44], [45]. Inactive Rad3-Rad26 complex was purified from an over-expression system in *S. pombe*. The kinase-inactive Cds1(D312E) purified from *E. coli* was used as the substrate [52]. When inactive Rad3-Rad26 was incubated with the substrate, a basal kinase activity was observed in phosphorylating Cds1-Thr^11^, the authentic Rad3 phosphorylation site [55]. This basal kinase activity of Rad3 is consistent with what has been observed *in vivo* [51], [52] and *in vitro* [55] and suggests that the active center of inactive Rad3 is in an intrinsically active conformation. As expected for a typical kinase reaction, phosphorylation of Cds1 increased during the time course of the incubation (Fig. 6A) and was dependent on the amounts of enzyme added to the reactions (Fig. 6B). Only minimal phosphorylation of Cds1 was observed with the similarly purified kinase-dead Rad3(D2249E)-Rad26, indicating that the phosphorylation was due to Rad3. When purified full-length Rad4 was added to the kinase reaction at a wide range of concentrations, no significant increase in the kinase activity of Rad3 could be observed (Fig. 6D). Similar to that observed with Crb2 [41], further increases of the Rad4 protein in the reaction had a slightly negative effect on the kinase activity. We also purified Rad4 C-terminus from amino acids 470 to 648 using a SUMO tag fused at the N-terminus and tested its potential effect on Rad3 activation. No stimulating effect was obtained. Similar negative results were obtained when purified Rad4 and Rad4 C-terminus were tested for activating budding yeast Mec1-Ddc2 *in vitro* [23], [44] (data now shown). Together, the *in vitro* biochemical studies showed that purified Rad4 had no or a minimal direct effect on Rad3 activation. This result is consistent with all *in vivo* data described above and suggests an evolutionary pressure for the convergence of functions in other eukaryotic organisms. However, the *in vitro* reactions lacking other known checkpoint components may be too simple to explain the physiological conditions inside the cells. Further *in vitro* biochemical studies that include the rest of the genetically defined components should provide a better explanation of how Rad3 is activated under various stress conditions in *S. pombe*.

## Discussion

The replication protein Rad4 is also known for its checkpoint function as a scaffold protein in response to DNA damage in *S. pombe*. Earlier reports have also suggested that Rad4 may function in the replication checkpoint when DNA replication is perturbed. Here, we show that unlike that in budding yeast Dpb11, the C-terminus of Rad4 does not contain a robust AAD required for Rad3 activation. Our genetic screen and the *in vitro* studies also suggested that as previously reported [59], the main function of Rad4 is to scaffold Crb2 and Rad9 for efficient activation of Chk1, but not Rad3. This conclusion is based on the following observations: First, the new C13Y and K56R mutations, which are believed to have eliminated most of the checkpoint function of Rad4, abolished the direct interaction with Crb2. Consequently, the mutations specifically blocked the activation of Chk1, but not Mrc1, Rad9, nor Cds1, by Rad3. Second, these two mutations showed a dramatic synthetic effect with Δ*cds1* but not with Δ*chk1* mutant when DNA damage occurs, indicating that Rad4 mainly function in the Chk1 pathway. Consistent with this, the relative sensitivities of all *rad4* mutants to HU and MMS were quite similar (Fig. 1D and 2B). Third, all mutations in C-terminus only slightly sensitized the cells to DNA damage and the mutant lacking the whole C-terminus was resistant to HU and MMS almost like the wild type cells. Importantly, phosphorylation of Cds1 and Chk1 and the immediate substrates Mrc1 and Rad9 by Rad3 was not affected by the C-terminal deletion. Finally, the negative results from the *in vitro* biochemical studies also support this conclusion. Since Rad4 had already been extensively screened [31], [33], [47], we believe that it is unlikely that a new mutant with defects only in the Cds1 pathway or Rad3 activation can be identified in Rad4.

### The sensitivity of Rad4 mutants to chronic HU treatment

It is remarkable that the C13Y and K56R mutants are highly sensitive to chronic exposure with low doses of HU (Fig. 2B) and yet resistant to acute HU treatment (Fig. 3C). In nature, yeast cells are continuously exposed to low levels of UV or other agents that perturb DNA replication [60], [61]. The acute treatments with high doses of UV, MMS or HU used in the laboratories can only happen in nature under extreme conditions. Chronic exposure to low doses of HU may be more relevant to yeast physiology. However, it is unclear how the replication checkpoint is activated and regulated under these conditions. Unlike the acute treatment with high doses of HU, which may arrest replication forks, chronic exposure to low doses of HU only slows the forks, which may potentially cause more DNA damage over time. Clearly, activation of the checkpoint and recovery from the checkpoint arrest need to be well coordinated under these situations [59], [62]. However, without knowing the number of dying cells and the amount of drug decomposing over time, the checkpoint activities are difficult to assess during a prolonged low dose HU treatment. Nevertheless, we believe that the sensitivity of these mutants to chronic HU treatment is caused by the complete elimination of Chk1 activity exacerbated by a possible minor defect in DNA replication.

### The scaffold function of Rad4

Rad4 is essential for its scaffold function in the initiation of DNA replication. The mechanistic details have been worked out in budding yeast and subsequently confirmed in fission yeast [38], [39], [40]. The protein binds to Sld2 and Sld3 that have been prephosphorylated by Cdc2(Cdc28) via its C- and N-terminal pairs of BRCT repeats, respectively, for stable loading and subsequent activation of replication helicase at the replication origins. Interestingly, the scaffold function of Rad4 is also required for checkpoint signaling although it is not completely clear how the scaffolding is regulated to coordinate the two separate functions of the protein. Since Rad4 interacts with phosphorylated Sld2, Sld3, and Rad9, it is believed that the interaction between Rad4 N-terminus with Crb2 is also phospho-dependent [63]. Consistently, our screened mutations are mainly located at the ionizable residues, especially the positively charged K56, which is thought to be in direct contact with a phosphorylated residue. Interestingly, the interaction between K56 and phosphorylated Crb2 may be more conformational than electrostatic in nature because the K56R and K56N mutants behaved the same as the charge reversal K56E mutant. Rad4 may also interact with other checkpoint proteins such as Rad3-Rad26, Mrc1 and Cdc2 [47], [48], [59]. It will be interesting to investigate the potential effects of the newly identified mutations on the protein-protein interactions.

### Activation mechanism of Rad3

The expression level of the ΔC mutant is higher than wild-type Rad4. This suggests that the C-terminus may facilitate the function of the N-terminal BRCT domains in scaffolding Sld2 and Sld3 under normal conditions. The higher level of the expressed ΔC protein may be required to compensate the potential weakening of the protein-protein interactions. The direct evidence that the point mutations in the C-terminus may minimally affect the replication function of Rad4 remains lacking. However, our preliminary data suggest this possibility that explains the observed minimal drug sensitivities and better cell survival from the HU-induced replication stress. Although it remains possible that the AAD in the Rad4 C-terminus may activate Rad3 under certain specific conditions or cell cycle stage [48], considering the drug resistance and the intact Rad3-dependent phosphorylation of multiple checkpoint proteins observed in the ΔC mutant, the intact Mrc1 phosphorylation by Rad3 in Δ*rad9* mutant [51], and the minimal effect of purified Rad4 and the C-terminus on Rad3 and Mec1 *in vitro*, we believe that unlike that in Dpb11, the C-terminus of Rad4 does not contain a robust AAD.

The exact mechanism of how Rad3, Mec1 and ATR are activated remains unknown because the crystal structures of these kinases are not yet available. The recently solved structures of a truncated mTOR, one of the Rad3 related PIKK kinases, may provide some implications [64]. The active center of mTOR kinase domain is in an intrinsically active conformation and activation of mTOR involves granting access of the active site to the substrates. The basal kinase activity observed in inactive Rad3 suggests that its kinase domain may also be in a constitutively active conformation. Since Rad3 is activated by unusually fast kinetics similar to ATM [25], it will be interesting to know how the basal kinase activity of Rad3 is further stimulated under various stress conditions. It is also possible that similar to that in budding yeast [65], [66], Rad3 can be activated by multiple redundant factors so that various substrates can be phosphorylated in response to different stresses. Interestingly, mutation of the potential AAD in fission yeast Rad9 (Ddc1 in *S. cerevisiae*) has only a minimal effect on checkpoint signaling [48]. Consistent with this, we have shown previously that phosphorylation of Mrc1 by Rad3 remains intact even in *rad9* null mutant under replication stress [51]. This suggests that Rad9 may not be the critical redundant factor required for direct Rad3 activation. It is possible that a new redundant factor remains to be identified in fission yeast that can robustly activate Rad3.

## Supporting Information

Figure S1 **Examination of temperature sensitivity of the newly identified** ***rad4*** **mutants with severe drug sensitivities.** Wild type *rad4^+^* or *rad4* with the newly identified mutations C13Y, K56R, and A60P in the first BRCT repeat were integrated at the *rad4^+^* genomic locus as described in Fig. 2A. After several steps of fivefold dilution, the cells were spotted on two YE6S plates. The two plates were separately incubated at 25°C or 37°C for three days and then photographed. Among the newly identified mutants, A60P is the only mutant that is sensitive to 37°C.
(PDF)

Figure S2 **The N-terminal C13Y and K56R mutations may have maximally eliminated the checkpoint function of Rad4.** (**A**) In order to search for an ideal *rad4* mutation that can completely separate the checkpoint function from the DNA replication function of Rad4, C13Y, K56R and the previously described E368K (Taricani and Wang 2006) mutations were combined to see whether the combined mutations can further increase the drug sensitivity. Combination of K56R with E368K is lethal, suggesting that the replication function is affected. The C13Y-K56R and C13Y-E368K mutants grow normally, however, no significant enhancement of drug sensitivity was observed. (**B**) Combination of F305S with E368K is lethal, consistent with the essential function of the third BRCT repeat in DNA replication. Together, these results showed that combinations of the point mutations cannot further eliminate the checkpoint function of Rad4. Since the C13Y and K56R mutants are even more sensitive to MMS than the Δ*chk1* mutant (Fig. 2B), they may have maximally eliminated the checkpoint function of Rad4.
(PDF)

Figure S3 **Phosphorylation of Mrc1-Thr^645^ by Rad3 was not affected by the Rad4 mutations.** Wild type cells or cells with the integrated *rad4* mutations were incubated with (+) or without (-) HU for three hours at 30°C. The cells were fixed in 15% TCA at 4°C for more than three hours. Whole cell lysates were made from the TCA-fixed cells by the mini-bead beater method. The samples were separated by SDS PAGE and transferred to a nitrocellulose membrane. The membrane was strained with Ponceau S. A section of the stained membrane is shown for the loading (lower panel). The top section of the membrane containing Mrc1 was cut out, destained, and blotted with phosphor-specific antibody against phosphorylated Mrc1-Thr^645^ (top panel). Asterisk indicates the cross-reactive material. The same membrane was stripped and reprobed with anti-Mrc1 antibodies to detect Mrc1 (middle panel). Under the replication stress induced by HU, phosphorylation of Mrc1-Thr^645^ by Rad3 remains intact in all tested *rad4* mutants.
(PDF)

Figure S4 **Time course study of the phosphorylation of Chk1 and Cds1-Thr^11^ by Rad3 in** ***rad4*** **mutants.** (**A**) Whole cell lysates made from MMS-treated cells under neutral conditions were separated by SDS PAGE and transferred to nitrocellulose membrane for Western blotting analysis. Phosphorylation of Chk1 was assessed by mobility shift assay using anti-HA antibody. A section of the Ponceau S stained membrane was shown below as the loading control. (**B**) Cds1 was IPed from the HU-treated cell and separated on an 8% SDS PAGE gel for Western blotting. Phosphorylated Cds1-Thr^11^ was detected by phospho-specific antibody (top). The same blot was stripped and reprobed with anti-HA antibody to detect Cds1 (bottom). IgG indicates the heavy chain of the anti-HA antibody used for the IP. This result ruled out the possibility of indirect cell cycle effect of the *rad4* mutations on the phosphorylation of Chk1 and Cds1.
(PDF)

Figure S5 **Phosphorylated Rad9-Thr^412^ by Rad3 recruits Rad4.** (**A**) Binding of Rad4 to Rad9 is dependent on the phosphorylation of Rad9-Thr^412^ by Rad3. Rad4 was Co-IPed with Rad9 from HU-treated cells using anti-HA antibody beads. The beads with the IPed proteins were then treated with λ phosphatase in the presence or absence of the phosphatase inhibitor. Samples in lanes 3 and 5 were similarly treated except the beads in lane 5 were washed once before the SDS PAGE analysis. The phosphatase treatment removed the phosphate group on Rad9-Thr^412^ (compare lanes 3 with lane 4), which eliminated the binding of Rad4 to the Rad9 (compare lane 3 with lane 5). (**B**) Mutations of the Rad9 phosphorylation sites have a stronger effect than the Rad4(E368K) mutation on Rad3 dependent phosphorylation of Cds1-Thr^11^. Cds1 was IPed from the Rad9 phosphorylation site T412A or T412A-S423A mutants or the Rad4(E368K) mutants or the double mutants containing both Rad9 and Rad4 mutations. Phosphorylation of Cds1-Thr^11^ was detected by Western blotting using the phospho-specific antibody and quantitated. The levels of Cds1-Thr^11^ phosphorylation are shown as percentages with that in HU-treated wild type cells being set as 100%. Loading of Cds1 was shown in the lower panel by Western blotting using anti-HA antibody. (**C**) Phosphorylation of Rad9 functions in the upstream of Rad4 recruitment in the Chk1-mediated DNA damage response. The phosphorylation site mutants of Rad9 T412A and T412A-S423A were crossed with the Rad4(E368K) mutant. The single and double mutants were tested for their sensitivities to MMS by spot assay. Since Rad4(E368K) mutation affects the interaction of Rad4 with phosphorylated Rad9 and the Rad9 phosphorylation site mutations have a dominant effect over the Rad4(E368K) mutation, phosphorylated Rad9 recruits Rad4 to the DNA damage sites for efficient activation of Chk1. Together, these results suggest that phosphorylation of Rad9 by Rad3 (or activation of Rad3) does not absolutely require the recruitment of Rad4.
(PDF)

Figure S6 **Minimal drug sensitivity caused by the C-terminal mutations and the dominant effect of the N-terminal mutations in the DNA damage response.** (**A**) Diagram of the Rad4 C-terminus with relative locations of the mutations. The enlarged portion contains the recently reported Y599R (open circle) in the AAD domain (underlined) (Lin *et al*. 2012) and the three point mutations isolated by this study (solid circles). ΔC is the mutant in which the whole C-terminus from amino acids 498 to 648 is deleted. (**B**) Minimal drug sensitivities of the C-terminal mutants. The C-terminal mutations, including the previously described Y599R (Lin *et al*. 2012) mutation, were all integrated at *rad4^+^* genomic locus as described in Fig 2 and tested for their sensitivities to HU and MMS. Asterisks indicate two previously described mutants (Lin *et al*. 2012) (strains SJ3 and SJ5) included in this experiment for comparison. (**C**) Dominant effects of the N-terminal mutation in the DNA damage checkpoint pathway. The N-terminal mutations C13Y and K56R (bold face) were combined with the C-terminal Y599R or D601N-K608E-L615S mutations and expressed on the vector under the control of *rad4^+^* promoter in the shut-off strain. The drug sensitivities of the indicated mutants were tested on plates containing 0.01% MMS or HU at 2.5 mM or 5 mM concentrations. When cells are treated with MMS or 5 mM HU, the N-terminal mutations have the dominant effect over the C-terminal mutations. However, under the moderate replication stress generated by HU at the low level of 2.5 mM, which may cause minimal DNA damage, the C-terminal mutations are dominant over the N-terminal mutations in promoting the cell survival.
(PDF)

Figure S7 **Minimal drug sensitivities caused by all mutations in the C-terminus of Rad4.** (**A**) Diagram of Rad4 with the relative locations of the four BRCT repeats (roman numerals) and the three point mutations D601N, K608E, and L615S newly identified by this study. A series of deletion mutations were also made that are indicated by the lines of various lengths. (**B**) The three new C-terminal mutations were integrated at the *rad4^+^* genomic locus. Various combinations of the three mutations were also integrated by the same method. Sensitivities of the cells with the integrated mutations to HU, MMS and UV were assessed by spot assay. Wild type cells and the Δ*rad3* mutant were used as the controls. The results showed that mutations in or near the recently identified AAD domain in the C-terminus only had a minor defect in checkpoint responses to DNA damage and the replication stress. (**C**) Wild type Rad4 or Rad4 with the indicated deletion mutations were expressed on a vector in the shut-off strain and tested for drug sensitivity. The three point mutations D601N, K698E, and L615S were included for comparison. Small deletions from the C-terminal ends did not affect much of the checkpoint functions except the Δ498–648 mutation, which generated a minimal sensitivity to MMS but not to HU similar to that in the three point mutations (top panel). The ΔC mutant, in which the whole C-terminus from amino acid 498 to 648 is deleted, behaved like the wild type cells. A larger deletion (Δ474–648) slightly sensitized the cells to HU but not MMS indicating a minor defect in DNA replication (lower panel). Further deletions into the third and fourth BRCT repeat (Δ376–648) and (Δ301–648) are lethal indicating that these repeats are required for DNA replication. (**D**) Mutations of the two tryptophan residues W485 and W580 in the C-terminus as well as the tryptophan W253 located between the third and the fourth BRCT repeats did not affect the checkpoint function of Rad4. (**E**) The protein levels of wild type Rad4 and Rad4 with various C-terminal mutations expressed on the vector in the shut-off strain were examined by Western blotting using anti-Rad4 antibodies. Asterisk indicates the cross-reactive material. All mutations in Rad4 C-terminus did not affect the level of the protein.
(PDF)

Table S1 **The** ***S. pombe*** **strains used in this study.**
(DOCX)
