# Correction: Rad4 Mainly Functions in Chk1-Mediated DNA Damage Checkpoint Pathway as a Scaffold Protein in the Fission Yeast *Schizosaccharomyces pombe*

**DOI:** 10.1371/journal.pone.0204070

**Published:** 2018-09-11

**Authors:** Ming Yue, Li Zeng, Amanpreet Singh, Yongjie Xu

The affiliation for the second author is incorrect. The correct affiliation for Li Zeng is Department of Clinical Laboratory, The First Affiliated Hospital of Zhengzhou University, Zhengzhou City, China.
