# Supplementary figures and images for: Rad4 Mainly Functions in Chk1-Mediated DNA Damage Checkpoint Pathway as a Scaffold Protein in the Fission Yeast Schizosaccharomyces pombe
Source: PLoS One. 2014 Mar 24;9(3):e92936. doi: 10.1371/journal.pone.0092936 (PMC3963969; doi:10.1371/journal.pone.0092936)

Figure S1

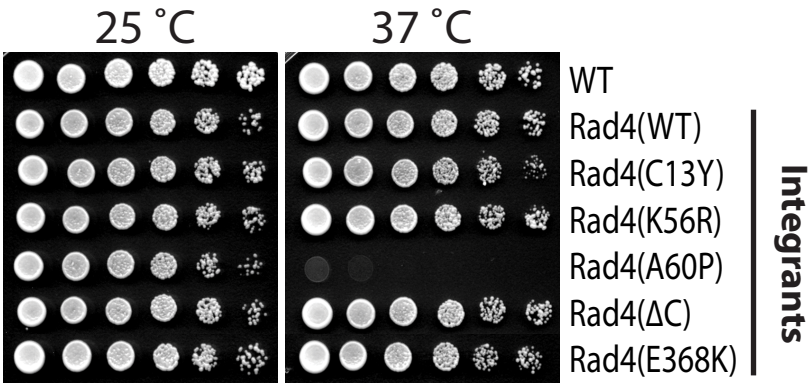

Supplement: Figure S1 — Examination of temperature sensitivity of the newly identified rad4 mutants with severe drug sensitivities. Wild type rad4+ or rad4 with the newly identified mutations C13Y, K56R, and A60P in the first BRCT repeat were integrated at the rad4+ genomic locus as described in Fig. 2A. After several steps of fivefold dilution, the cells were spotted on two YE6S plates. The two plates were separately incubated at 25°C or 37°C for three days and then photographed. Among the newly identified mutants, A60P is the only mutant that is sensitive to 37°C. (PDF) [file pone.0092936.s001.pdf]

Figure S2

A

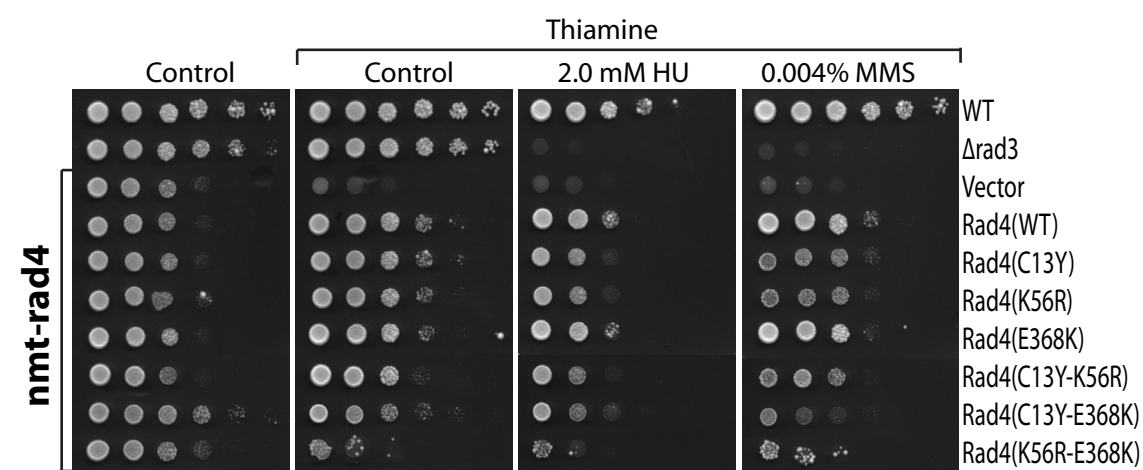

B

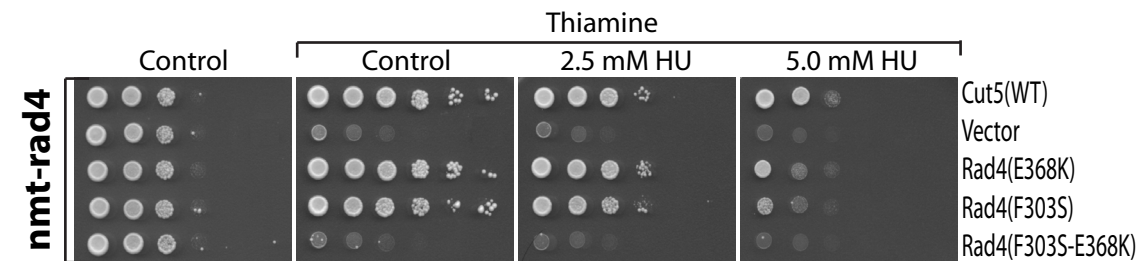

Supplement: Figure S2 — The N-terminal C13Y and K56R mutations may have maximally eliminated the checkpoint function of Rad4. (A) In order to search for an ideal rad4 mutation that can completely separate the checkpoint function from the DNA replication function of Rad4, C13Y, K56R and the previously described E368K (Taricani and Wang 2006) mutations were combined to see whether the combined mutations can further increase the drug sensitivity. Combination of K56R with E368K is lethal, suggesting that the replication function is affected. The C13Y-K56R and C13Y-E368K mutants grow normally, however, no significant enhancement of drug sensitivity was observed. (B) Combination of F305S with E368K is lethal, consistent with the essential function of the third BRCT repeat in DNA replication. Together, these results showed that combinations of the point mutations cannot further eliminate the checkpoint function of Rad4. Since the C13Y and K56R mutants are even more sensitive to MMS than the Δchk1 mutant (Fig. 2B), they may have maximally eliminated the checkpoint function of Rad4. (PDF) [file pone.0092936.s002.pdf]

Figure S3

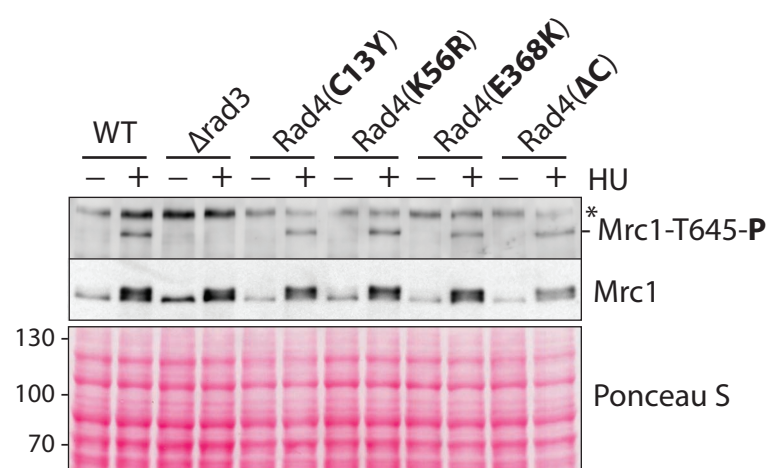

Supplement: Figure S3 — Phosphorylation of Mrc1-Thr645 by Rad3 was not affected by the Rad4 mutations. Wild type cells or cells with the integrated rad4 mutations were incubated with (+) or without (-) HU for three hours at 30°C. The cells were fixed in 15% TCA at 4°C for more than three hours. Whole cell lysates were made from the TCA-fixed cells by the mini-bead beater method. The samples were separated by SDS PAGE and transferred to a nitrocellulose membrane. The membrane was strained with Ponceau S. A section of the stained membrane is shown for the loading (lower panel). The top section of the membrane containing Mrc1 was cut out, destained, and blotted with phosphor-specific antibody against phosphorylated Mrc1-Thr645 (top panel). Asterisk indicates the cross-reactive material. The same membrane was stripped and reprobed with anti-Mrc1 antibodies to detect Mrc1 (middle panel). Under the replication stress induced by HU, phosphorylation of Mrc1-Thr645 by Rad3 remains intact in all tested rad4 mutants. (PDF) [file pone.0092936.s003.pdf]

Figure S4

A

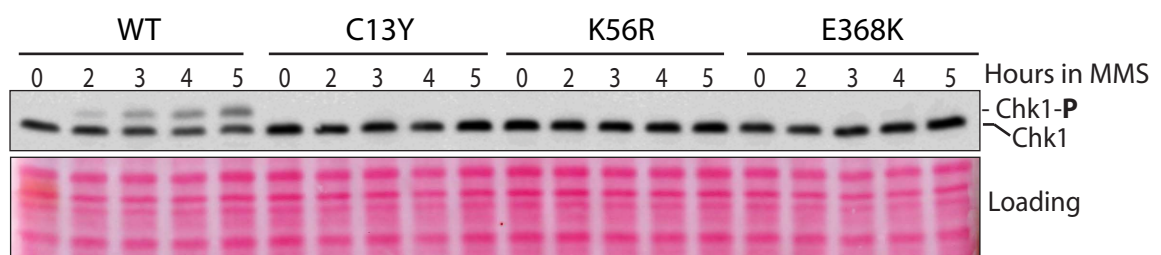

B

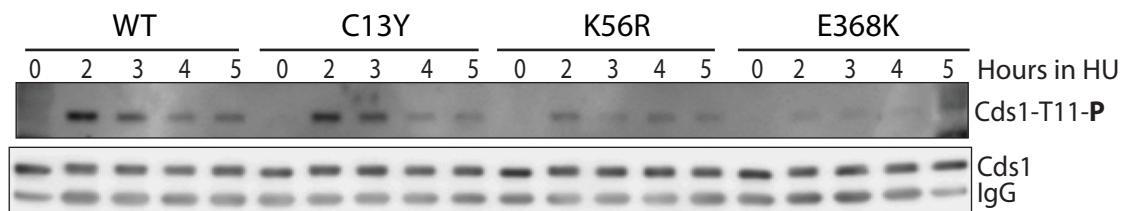

Supplement: Figure S4 — Time course study of the phosphorylation of Chk1 and Cds1-Thr11 by Rad3 in rad4 mutants. (A) Whole cell lysates made from MMS-treated cells under neutral conditions were separated by SDS PAGE and transferred to nitrocellulose membrane for Western blotting analysis. Phosphorylation of Chk1 was assessed by mobility shift assay using anti-HA antibody. A section of the Ponceau S stained membrane was shown below as the loading control. (B) Cds1 was IPed from the HU-treated cell and separated on an 8% SDS PAGE gel for Western blotting. Phosphorylated Cds1-Thr11 was detected by phospho-specific antibody (top). The same blot was stripped and reprobed with anti-HA antibody to detect Cds1 (bottom). IgG indicates the heavy chain of the anti-HA antibody used for the IP. This result ruled out the possibility of indirect cell cycle effect of the rad4 mutations on the phosphorylation of Chk1 and Cds1. (PDF) [file pone.0092936.s004.pdf]

Figure S5

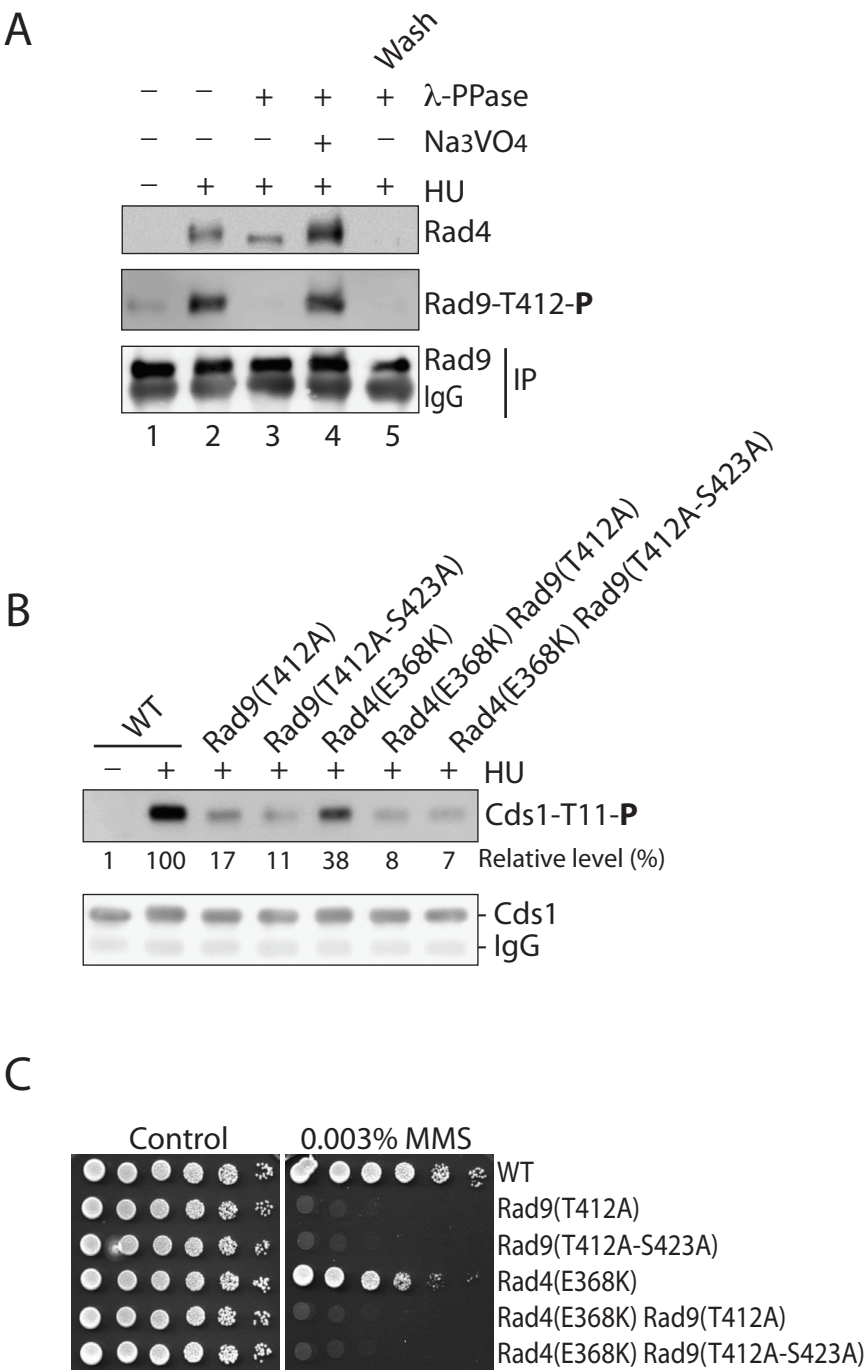

Supplement: Figure S5 — Phosphorylated Rad9-Thr412 by Rad3 recruits Rad4. (A) Binding of Rad4 to Rad9 is dependent on the phosphorylation of Rad9-Thr412 by Rad3. Rad4 was Co-IPed with Rad9 from HU-treated cells using anti-HA antibody beads. The beads with the IPed proteins were then treated with λ phosphatase in the presence or absence of the phosphatase inhibitor. Samples in lanes 3 and 5 were similarly treated except the beads in lane 5 were washed once before the SDS PAGE analysis. The phosphatase treatment removed the phosphate group on Rad9-Thr412 (compare lanes 3 with lane 4), which eliminated the binding of Rad4 to the Rad9 (compare lane 3 with lane 5). (B) Mutations of the Rad9 phosphorylation sites have a stronger effect than the Rad4(E368K) mutation on Rad3 dependent phosphorylation of Cds1-Thr11. Cds1 was IPed from the Rad9 phosphorylation site T412A or T412A-S423A mutants or the Rad4(E368K) mutants or the double mutants containing both Rad9 and Rad4 mutations. Phosphorylation of Cds1-Thr11 was detected by Western blotting using the phospho-specific antibody and quantitated. The levels of Cds1-Thr11 phosphorylation are shown as percentages with that in HU-treated wild type cells being set as 100%. Loading of Cds1 was shown in the lower panel by Western blotting using anti-HA antibody. (C) Phosphorylation of Rad9 functions in the upstream of Rad4 recruitment in the Chk1-mediated DNA damage response. The phosphorylation site mutants of Rad9 T412A and T412A-S423A were crossed with the Rad4(E368K) mutant. The single and double mutants were tested for their sensitivities to MMS by spot assay. Since Rad4(E368K) mutation affects the interaction of Rad4 with phosphorylated Rad9 and the Rad9 phosphorylation site mutations have a dominant effect over the Rad4(E368K) mutation, phosphorylated Rad9 recruits Rad4 to the DNA damage sites for efficient activation of Chk1. Together, these results suggest that phosphorylation of Rad9 by Rad3 (or activation of Rad3) does not absolutely require the [file pone.0092936.s005.pdf]

Figure S6

A

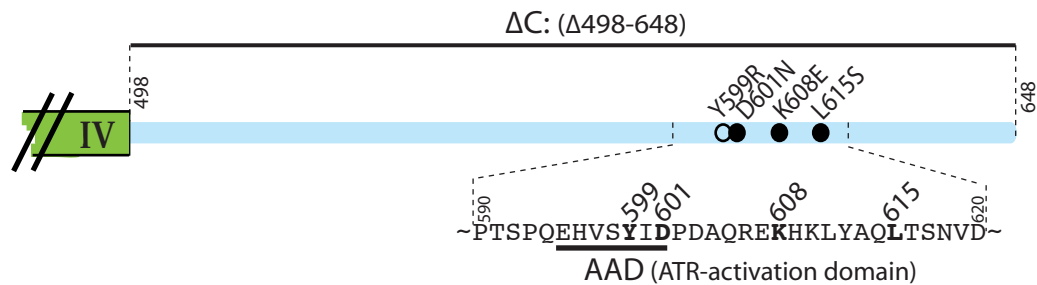

B

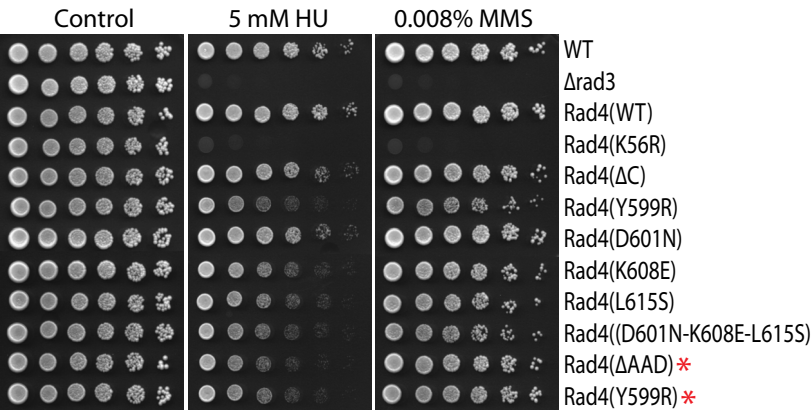

C

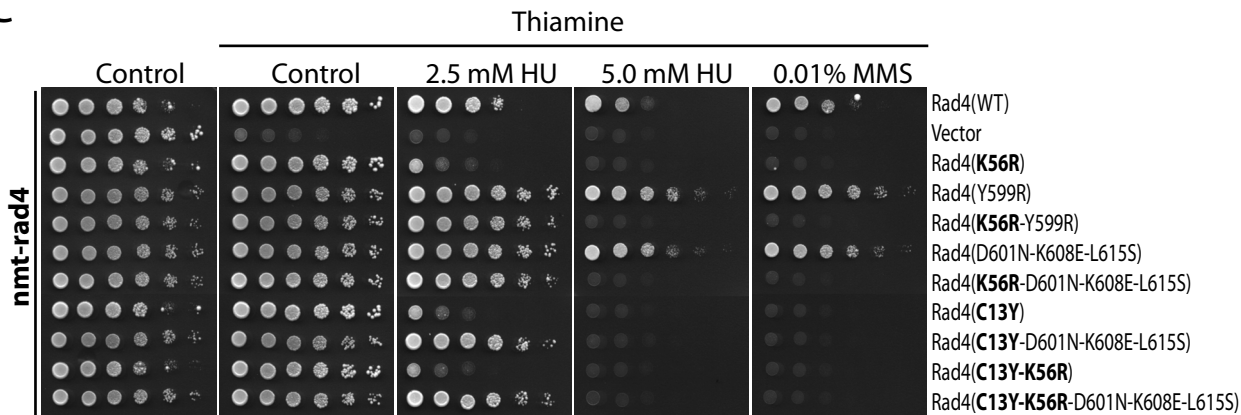

Supplement: Figure S6 — Minimal drug sensitivity caused by the C-terminal mutations and the dominant effect of the N-terminal mutations in the DNA damage response. (A) Diagram of the Rad4 C-terminus with relative locations of the mutations. The enlarged portion contains the recently reported Y599R (open circle) in the AAD domain (underlined) (Lin et al. 2012) and the three point mutations isolated by this study (solid circles). ΔC is the mutant in which the whole C-terminus from amino acids 498 to 648 is deleted. (B) Minimal drug sensitivities of the C-terminal mutants. The C-terminal mutations, including the previously described Y599R (Lin et al. 2012) mutation, were all integrated at rad4+ genomic locus as described in Fig 2 and tested for their sensitivities to HU and MMS. Asterisks indicate two previously described mutants (Lin et al. 2012) (strains SJ3 and SJ5) included in this experiment for comparison. (C) Dominant effects of the N-terminal mutation in the DNA damage checkpoint pathway. The N-terminal mutations C13Y and K56R (bold face) were combined with the C-terminal Y599R or D601N-K608E-L615S mutations and expressed on the vector under the control of rad4+ promoter in the shut-off strain. The drug sensitivities of the indicated mutants were tested on plates containing 0.01% MMS or HU at 2.5 mM or 5 mM concentrations. When cells are treated with MMS or 5 mM HU, the N-terminal mutations have the dominant effect over the C-terminal mutations. However, under the moderate replication stress generated by HU at the low level of 2.5 mM, which may cause minimal DNA damage, the C-terminal mutations are dominant over the N-terminal mutations in promoting the cell survival. (PDF) [file pone.0092936.s006.pdf]

Figure S7

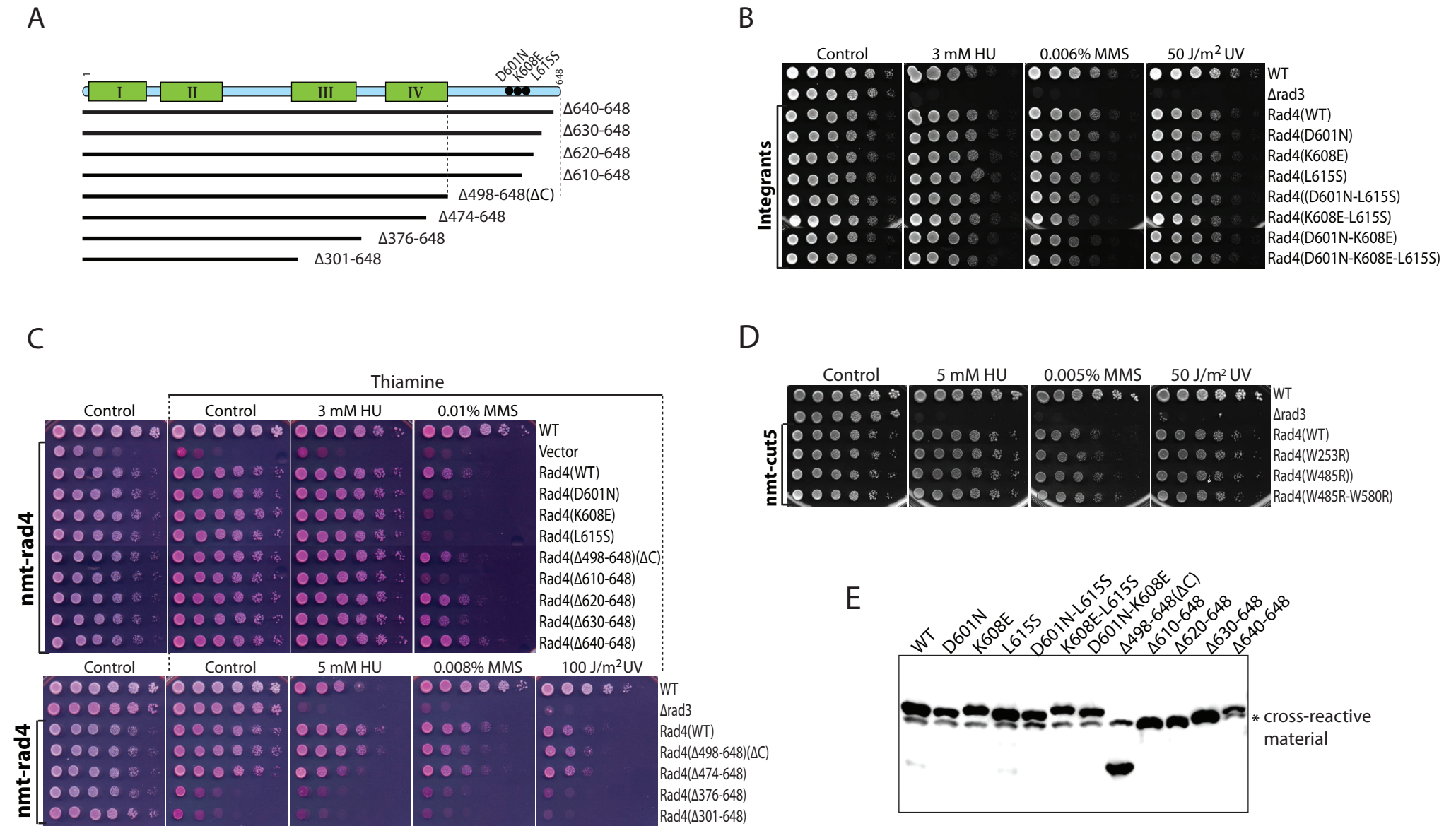

Supplement: Figure S7 — Minimal drug sensitivities caused by all mutations in the C-terminus of Rad4. (A) Diagram of Rad4 with the relative locations of the four BRCT repeats (roman numerals) and the three point mutations D601N, K608E, and L615S newly identified by this study. A series of deletion mutations were also made that are indicated by the lines of various lengths. (B) The three new C-terminal mutations were integrated at the rad4+ genomic locus. Various combinations of the three mutations were also integrated by the same method. Sensitivities of the cells with the integrated mutations to HU, MMS and UV were assessed by spot assay. Wild type cells and the Δrad3 mutant were used as the controls. The results showed that mutations in or near the recently identified AAD domain in the C-terminus only had a minor defect in checkpoint responses to DNA damage and the replication stress. (C) Wild type Rad4 or Rad4 with the indicated deletion mutations were expressed on a vector in the shut-off strain and tested for drug sensitivity. The three point mutations D601N, K698E, and L615S were included for comparison. Small deletions from the C-terminal ends did not affect much of the checkpoint functions except the Δ498–648 mutation, which generated a minimal sensitivity to MMS but not to HU similar to that in the three point mutations (top panel). The ΔC mutant, in which the whole C-terminus from amino acid 498 to 648 is deleted, behaved like the wild type cells. A larger deletion (Δ474–648) slightly sensitized the cells to HU but not MMS indicating a minor defect in DNA replication (lower panel). Further deletions into the third and fourth BRCT repeat (Δ376–648) and (Δ301–648) are lethal indicating that these repeats are required for DNA replication. (D) Mutations of the two tryptophan residues W485 and W580 in the C-terminus as well as the tryptophan W253 located between the third and the fourth BRCT repeats did not affect the checkpoint function of Rad4. (E) The protein levels of wild type Ra [file pone.0092936.s007.pdf]
