# Supplementary material for: Rad4 Mainly Functions in Chk1-Mediated DNA Damage Checkpoint Pathway as a Scaffold Protein in the Fission Yeast Schizosaccharomyces pombe
Source: PLoS One. 2014 Mar 24;9(3):e92936. doi: 10.1371/journal.pone.0092936 (PMC3963969; doi:10.1371/journal.pone.0092936)
Supplement: Table S1 — The S. pombe strains used in this study. (DOCX) [file pone.0092936.s008.docx]

| **Strain** | **Genotype** | **Source** |
| --- | --- | --- |
| YJ945 | *nmt81-rad4*^+^ *leu1-32 ura4-D18 ade6* | This study |
| YM248 | *rad4:LEU2 ura4-D18 ade6* | This study |
| YM251 | *rad4(K56R):LEU2 ura4-D18 ade6* | This study |
| YM253 | *rad4(A60P):LEU2 ura4-D18 ade6* | This study |
| YM254 | *rad4(C13Y):LEU2 ura4-D18 ade6* | This study |
| YM257 | *rad4(E368K):LEU2 ura4-D18 ade6* | This study |
| YM279 | *rad4(ΔC):LEU2 ura4-D18 ade6* | This study |
| YM400 | *rad4:LEU2 cds1-6His2HA ura4-D18 ade6* | This study |
| YM401 | *rad4(C13Y):LEU2 cds1-6His2HA ura4-D18 ade6* | This study |
| YM402 | *rad4(K56R):LEU2 cds1-6His2HA ura4-D18 ade6* | This study |
| YM493 | *rad4(E368K):LEU2 cds1-6His2HA ura4-D18 ade6* | This study |
| YM424 | *rad4(ΔC):LEU2 cds1-6His2HA ura4-D18 ade6* | This study |
| YM513 | *rad4:LEU2 chk1-9myc2HA6His:ura4^+^ ura4-D18* | This study |
| YM514 | *rad4(C13Y):LEU2 chk1-9myc2HA6His:ura4^+^ ura4-D18* | This study |
| YM515 | *rad4(K56R):LEU2 chk1-9myc2HA6His:ura4^+^ ura4-D18* | This study |
| YM517 | *rad4(E368K):LEU2 chk1-9myc2HA6His:ura4^+^ ura4-D18* | This study |
| YM518 | *rad4(ΔC):LEU2 chk1-9myc2HA6His:ura4^+^ ura4-D18* | This study |
| YM519 | *rad4:LEU2 ∆rad9::ura4^+^ leu:3HA-rad9 ade6* | This study |
| YM520 | *rad4(C13Y):LEU2 ∆rad9::ura4^+^leu1:3HA-rad9 ade6* | This study |
| YM521 | *rad4(K56R):LEU2 ∆rad9::ura4^+^leu1:3HA-rad9 ade6* | This study |
| YM523 | *rad4(E368K):LEU2 ∆rad9::ura4^+^leu1:3HA-rad9 ade6* | This study |
| YM524 | *rad4(ΔC):LEU2 ∆rad9::ura4^+^leu1:3HA-rad9 ade6* | This study |
| YM564 | *rad4(C13Y+K56R):LEU2 ∆rad9::ura4^+^leu1:3HA-rad9 ade6* | This study |
| YM607 | *rad4:LEU2 ∆rad9::ura4^+^leu1:3HA-rad9(T412A+S423A) ade6* | This study |
| YM608 | *rad4(E368K):LEU2 ∆rad9::ura4^+^leu1:3HA-rad9(T412A+S423A) ade6M210/M216* | This study |
| TK48 | *leu1-32 ade6* | Kelly lab |
| NR1826 | *∆rad3::ura4^+^ leu1-32 ade6* | Russell lab |
| GBY191 | *∆cds1::ura4^+^ leu1-32 ade6* | Lab Stock |
| TK197 | *∆chk1::ura4^+^ leu1-32 ade6* | Kelly lab |
| YJ374 | *cds1-6His2HA leu1-32 ura4-D18 ade6* | Lab Stock |
| YJ795  YJ1344  SJ3  SJ5 | *∆rad9::ura4^+^ leu1:3HA-rad9 ura4-D18 ade6-704*  *rad4(Y599R):LEU2 ura4-D18 ade6*  *loxP-rad4(∆AAD)-loxM3 ura4-D18 leu1-32 ade6-704*  *loxP-rad4(Y599R)-loxM3 ura4-D18 leu1-32 ade6-704* | Lab Stock  This study  Carr lab  Carr lab |
